# Proteomic Analysis of the Differential Response of *Pseudomonas aeruginosa* and *Staphylococcus aureus* to *Lacticaseibacillus rhamnosus* Cell-Free Supernatant and Lactic Acid

**DOI:** 10.3390/antibiotics14121271

**Published:** 2025-12-15

**Authors:** Marta Bianchi, Giuseppantonio Maisetta, Semih Esin, Giovanna Batoni, Kevin Kavanagh

**Affiliations:** 1 Department of Translational Research and New Technologies in Medicine and Surgery, University of Pisa, Via S. Zeno 37, 56123 Pisa, Italy; giuseppantonio.maisetta@unipi.it (G.M.); semih.esin@unipi.it (S.E.); 2 Department of Biology, Maynooth University, W23 F2K8 Maynooth, Ireland; kevin.kavanagh@mu.ie

**Keywords:** probiotics, postbiotics, antimicrobials, cell-free supernatants, *L. rhamnosus*, *P. aeruginosa*, *S. aureus*, proteomics

## Abstract

**Background/Objectives:** Postbiotics derived from lactic acid bacteria are emerging as promising antimicrobial agents due to their antibacterial, antibiofilm, and immunomodulatory properties. Among their metabolites, lactic acid (LA) is thought to play a major role in antimicrobial activity. This study investigated the proteomic response of *Pseudomonas aeruginosa* and *Staphylococcus aureus* to *Lacticaseibacillus rhamnosus* cell-free supernatant (CFS) and compared it with that elicited by LA alone. **Methods:** Overnight bacterial cultures were exposed to sub-MIC LA or CFS (1:10 for *P. aeruginosa* and 1:8 for *S. aureus*; ~12.5–15.6 mM LA) for 6 h at 37 °C. Intracellular proteins were harvested and subsequently quantified and purified to be analysed by HPLC–MS/MS, for quantitative label-free proteomics. **Results:** Proteomic analysis revealed clear separation of treated samples from controls, with largely overlapping responses to CFS and LA. Hallmark acid-stress adaptations were observed, including urease-mediated pH buffering, confirming that part of the response was driven by mild organic acid. In *P. aeruginosa*, treatments suppressed virulence pathways (phenazines, T3SS), while shifting metabolism toward lactate utilisation and reinforcing the outer membrane (lipid A, polyamine). In *S. aureus*, decreased abundance of the SaeRS-regulated immune-evasion factor Sbi, together with changes in envelope, ROS and translation-related proteins, suggested a bacteriostatic-like state. *S. aureus* differences between CFS and LA were more pronounced; CFS uniquely increased cell-wall defences, oxidative stress (SodA, SodM) and chaperone expression (GroS, GrpE), suggesting stress beyond acidification alone. **Conclusions:** These findings shed light on the molecular mechanisms underlying bacterial adaptation to CFS and highlight their potential as a novel antimicrobial approach.

## 1. Introduction

Antimicrobial resistance represents one of the most pressing challenges in modern medicine, contributing to an estimated 4.95 million deaths globally in 2021, with 1.14 million deaths directly attributable to drug-resistant bacterial infections [1]. *Pseudomonas aeruginosa* and *Staphylococcus aureus* are key contributors to this crisis, owing to their remarkable ability to develop antibiotic resistance, adapt to diverse environments, and cause a broad spectrum of human infections, particularly in hospital settings and among immunocompromised individuals [2]. Both species are currently classified by the World Health Organisation (WHO) as high-priority pathogens for which research and development of new antimicrobial strategies, complementing or replacing conventional antibiotics, are urgently needed [3].

The rapidly expanding field of probiotics and their derivatives is currently attracting significant interest for its potential to address antibiotic resistance. Emerging evidence supports their use not only as dietary supplements, but also as therapeutic agents in the management of drug-resistant bacterial infections and other human disorders [4,5]. In this regard, a growing body of research highlights the efficacy of probiotics, particularly lactic acid bacteria (LAB), in inhibiting pathogens’ growth and virulence through multiple mechanisms. These include competition for nutrients and adhesion sites, enhancement of host immunity, production of antimicrobial metabolites such as organic acids and bacteriocins, and interference with metabolic and signalling pathways of pathogenic bacteria [6,7]. Interestingly, many of these effects are preserved in probiotic-derived inanimate preparations, known as “postbiotics”, which may offer enhanced safety, particularly for immunocompromised individuals, as well as improved shelf stability and easier production compared to live probiotics [8,9,10,11].

Beyond these theoretical and technological advantages, preclinical evidence supports the feasibility of postbiotic-based interventions in vivo, both for preventing and managing infection-related conditions. For example, Abán and colleagues demonstrated that nebulised CFS from *Lactiplantibacillus plantarum* can be safely delivered to the lungs in a murine model of *P. aeruginosa* pneumonia, leading to significant reductions in bacterial burden and lung inflammation [12]. Additional support for the translational potential of LAB-derived therapies comes from earlier studies showing that intratracheal or intranasal administration of live lactobacilli protects mice from *P. aeruginosa* pneumonia by enhancing bacterial clearance and modulating airway inflammatory responses [13]. More recent formulation studies also reinforce the technological and biological plausibility of LAB-based inhalation strategies [14,15].

Bioactive compounds secreted into the extracellular environment during the growth and fermentation of food ingredients or complex microbiological media (e.g., De Man, Rogosa and Sharpe Broth, MRSB) are responsible for many of the direct antimicrobial effects attributed to probiotics [16,17]. In line with this, the bioactivity of *L. rhamnosus*–derived CFS reflects a multifactorial mixture of secreted metabolites, several of which we and others have partially characterised. In our recent work [18], the CFS was shown to contain high lactic acid concentrations and an acidic pH closely linked to its antimicrobial activity over time, together with a diverse pool of proteinaceous components identified through proteomic analysis. Size-based fractionation at 3 kDa indicated that most of the direct antibacterial effect resides in the ≤3 kDa fraction, which is enriched in lactic acid. Nevertheless, the unfractionated CFS still displayed a stronger inhibitory effect than this fraction alone, suggesting that additional non-acidic components may also contribute to the overall activity [18]. Consistently, quantitative proteomic analysis of *L. rhamnosus* CFS revealed the presence of several high-molecular-weight proteins with predicted antibacterial or antibiofilm functions, supporting their potential role as complementary effectors within the multifactorial activity of the whole supernatant [18]. Some of these effects may be pH-dependent, likely driven by lactic acid (LA) production, while others persist after pH neutralisation, suggesting the involvement of additional bioactive molecules such as bacteriocins. For example, cell-free supernatants derived from the newly isolated *L. rhamnosus* strain A5 [16], retain antimicrobial activity under acidic, thermal, and UV-stress conditions, but such activity is abolished by protease treatment, confirming the presence of heat-stable, proteinaceous antimicrobials, particularly bacteriocins that were effective against both Gram-positive and Gram-negative bacteria, including *S. aureus* and *Escherichia coli* [16].

In addition to bacteriocins, *L. rhamnosus* and related *LAB* are known to secrete multiple classes of bioactive molecules, including organic acids and other small-molecule postbiotics, as well as polymeric and surface-active factors such as exopolysaccharides (EPS) and biosurfactants [19,20,21]. *L. rhamnosus* produces biosurfactants with potent antibiofilm and antibacterial effects, particularly against methicillin-resistant *S. aureus* (MRSA). These biosurfactants disrupt biofilms through membrane destabilisation and interference with bacterial adhesion processes [22], offering an additional mechanism by which postbiotics may control biofilm-associated infections (e.g., chronic wound and device-associated infections). Together, these components form a multifactorial antimicrobial repertoire in which acidic and higher-molecular-weight elements are likely to act in combination to inhibit pathogenic bacteria.

A detailed analysis of the effects of cell-free supernatants from LAB on pathogenic microorganisms could help clarify the health-promoting role of probiotics and support the development of probiotic-derived inanimate preparations as antimicrobials.

Among LAB, *Lacticaseibacillus rhamnosus* is one of the most well-characterised and widely used probiotic strains [23]. Formerly classified as *Lactobacillus rhamnosus*, it was reclassified based on genetic analyses that distinguished it from closely related species [24]. In particular, *L. rhamnosus* GG (ATCC 53103) is known for its resistance to gastrointestinal stress and its ability to adhere to intestinal mucosa [25]. This strain has been extensively researched for its health benefits, including modulation of the gut microbiota, enhancement of immune responses, and prevention or treatment of gastrointestinal, respiratory, urogenital and otitis media infections [26,27,28,29]. *L. rhamnosus* is also used in food technology, particularly in dairy products and fermented foods, due to its ability to survive harsh processing conditions and contribute to flavour and texture [30].

Beyond antimicrobial effects, CFS from *L. rhamnosus* exhibit immunomodulatory properties critical for managing infection-driven inflammation. In macrophage models, they significantly reduced the secretion of pro-inflammatory cytokines such as TNF-α and IL-6, and decreased the levels of inducible enzymes like iNOS and COX-2, suggesting an anti-inflammatory profile that could help limit tissue damage during infection [31]. Likewise, recent findings demonstrated that *L. rhamnosus* CFS exert strong anti-inflammatory effects against human peripheral blood mononuclear cells (PBMCs) stimulated with LPS or biofilm-derived *P. aeruginosa* [18].

Since host–cell tolerance is central to their potential use, the cytotoxicity of *L. rhamnosus* CFS has been previously assessed in epithelial and immune cell models [18,32,33]. These studies indicate that the preparations are generally tolerated at the dilutions displaying antimicrobial or immunomodulatory activity, although the therapeutic window is relatively narrow and tolerance varies across cell types. Further work is therefore needed to clarify the conditions under which lactobacilli-derived supernatants may exert cytotoxic effects and to define strategies to minimise potential host–cell impact.

In a recent study, we identified the cell-free supernatants of *L. rhamnosus* (CFS) as one of the most active among LAB against clinical isolates of *P. aeruginosa* from cystic fibrosis [34]. The CFS also exhibited strong antibacterial activity in an in vitro wound infection model, significantly reducing both mono- and dual-species biofilms of *P. aeruginosa* and *S. aureus*, while avoiding the induction of antibiotic resistance [33]. Finally, the same CFS was demonstrated to be highly active against clinical isolates of *P. aeruginosa* in an in vitro lung infection model at the air-liquid interface, further supporting its development as an innovative antimicrobial [35].

Proteomic analysis has become an essential methodology for understanding how bacterial pathogens respond to environmental stressors, antibiotics, and host interactions at the molecular level [36,37]. Proteomics offers several key advantages over genomics and transcriptomics, as proteins are the primary effectors of bacterial cell processes, whereas genes and messenger RNA (mRNA) function as upstream regulatory elements. Consequently, proteomics offers a more direct and functionally relevant representation of cellular activity in response to stimuli, compared to genomic or transcriptomic analyses [38,39].

Label-free quantitative (LFQ) proteomics has demonstrated that *P. aeruginosa* regulates its proteome in response to environmental stresses or antibiotics; specifically, exposure to ciprofloxacin induces distinct protein expression patterns depending on the strain and treatment conditions, affecting pathways involved in resistance, biofilm formation, and oxidative stress response [40]. Similarly, *S. aureus* has been investigated using label-free proteomics to study its response to antibiotics; for example, when exposed to β-lactam antibiotics such as oxacillin, it revealed upregulation of resistance proteins such as penicillin-binding protein 2a, along with increased activity in peptidoglycan biosynthesis and coenzyme A metabolism, suggesting complex adaptation mechanisms to β-lactam stress [41]. Proteomics has also been successfully employed to assess the response of *S. aureus* and *P. aeruginosa* to unconventional antimicrobials, such as silver (I) acetate complex [42], further supporting the technique’s potential in elucidating pathogen responses to a broad spectrum of antimicrobial agents.

This study aimed to investigate how the cell-free supernatant of *L. rhamnosus* affects the proteome of *P. aeruginosa* and *S. aureus*. To determine whether the observed effects were due to the overall composition of the CFS or specifically to its LA content, we performed LFQ proteomics and compared the proteomic responses of *P. aeruginosa* and *S. aureus* treated with CFS to those treated with LA at the same concentration found in the supernatant. The results obtained demonstrated that the two bacterial species exhibit both similarities and differences in their adaptive proteomic responses to CFS and LA, respectively, offering insights into the mechanisms of action of CFS and their potential as innovative antimicrobial agents.

## 2. Results

### 2.1. Analysis of the Effects of CFS and LA on the Proteome of P. aeruginosa

#### 2.1.1. Proteomic Characterisation of *P. aeruginosa*

To assess how exposure to CFS and LA affects the proteome of *P. aeruginosa*, we carried out LFQ proteomic analysis of treated and untreated bacteria. Preliminary growth assessments were performed by measuring the OD_600_ of the treated cultures at the chosen time point of 6 h. The concentration of CFS used for *P. aeruginosa* was 1:10, corresponding to sub-inhibitory concentrations, which allows measurable stress responses without extensive loss of viability (Supplementary Figure S1). A total of 2921 proteins were initially identified through proteomic analysis. After filtering out contaminants and peptides identified by site, 2864 proteins remained. Post-imputation ANOVA (*p* < 0.05, fold change > 1.5) identified 378 proteins whose abundance differed significantly in at least one of the three experimental groups (CTRL, CFS, LA) (Supplementary Table S1).

**Figure 1:**
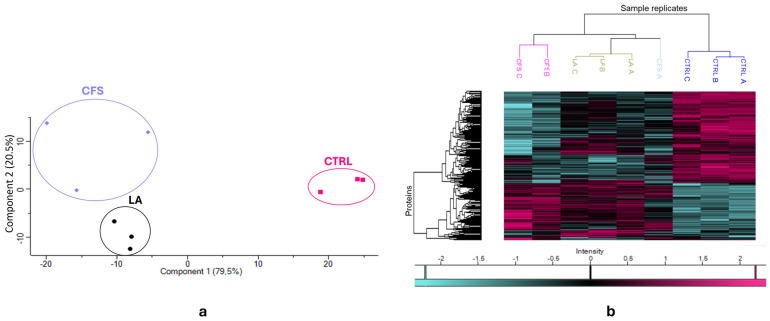
Principal components analysis (PCA) (**a**) and Heatmap (**b**) of the proteome of *P. aeruginosa* exposed to *L. rhamnosus* cell-free supernatant (CFS) and lactic acid (LA) as compared to the untreated control (CTRL).

Principal component analysis (PCA) of the *P. aeruginosa* dataset (Figure 1a) showed a pronounced separation of the control group (CTRL) from both treatment groups (CFS and LA), particularly along principal component 1. At the same time, CFS and LA are clustered closer to each other, indicating a higher degree of similarity between their proteomic profiles. This clustering pattern observed in the PCA is further supported by the heatmap analysis (Figure 1b), which also highlights the distinct proteomic signature of the CTRL group compared to the treated samples. The treated samples appear to be more similar to each other, with the CFS A sample clustering separately from the other two CFS samples in the dendrogram.

#### 2.1.2. Differential Proteomic Analysis of *P. aeruginosa* Following Exposure to CFS and LA

To investigate the impact of CFS and LA treatments on the *P. aeruginosa* proteome, volcano plots were generated using pairwise Student’s *t*-tests (*p* < 0.05) to identify differences in protein abundance between samples and to visualise changes in the associated pathways and biological processes.

**Figure 2:**
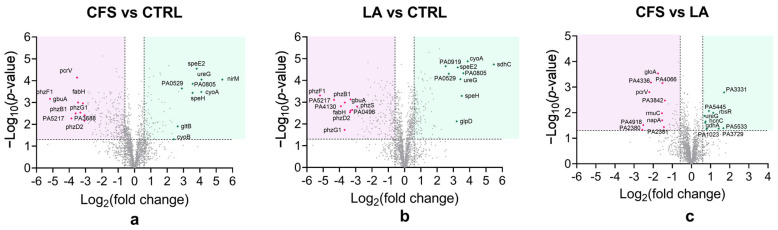
Volcano Plots of differentially expressed proteins in *P. aeruginosa*, showing the distribution of proteins based on statistical significance (−Log10 *p*-value) and magnitude of change (Log_2_ fold change) in the proteome of *P. aeruginosa* exposed to *L. rhamnosus* cell-free supernatant (CFS) compared to the untreated control (CTRL) (**a**), lactic acid (LA) compared to CTRL (**b**) and CFS compared to LA (**c**). Proteins above the significance threshold (*p* < 0.05) are considered differentially expressed. The top 10 most abundant proteins are highlighted in green, while the top 10 least abundant proteins are highlighted in pink. Gene names of related proteins are indicated next to each highlighted protein.

The plots (Figure 2a–c) illustrate the distribution of proteins based on statistical significance (−Log_10_ *p*-value) and magnitude of change (Log_2_ fold change) for the three pairwise comparisons: CFS versus untreated control (CTRL), LA versus CTRL, and LA versus CFS. Proteins above the significance threshold were considered differentially expressed.

For all comparisons, the ten most abundant proteins are highlighted in green and the ten least abundant proteins in pink. The proteins corresponding to the CFS vs. CTRL (Figure 2a) comparison are displayed in Table 1.

Among the top 10 most abundant *P. aeruginosa* proteins in the CFS-treated condition compared to the untreated control (Table 1), the most significantly abundant protein was Cytochrome c-551 (*nirM*; P00099), with a Log fold change of 5.38 and a Log10 (*p*-value) of 4.05. This was followed by Urease accessory protein UreG (*ureG*; Q9HUS0) and Cytochrome bo(3) ubiquinol oxidase subunit 2 (*cyoA*; Q9I427), with Log fold changes of 4.10 and 4.09, respectively. Other highly abundant proteins included Polyamine aminopropyltransferase 2 (*speE2*; Q9HV34; Log fold change: 3.81), DUF3509 domain-containing protein (*PA0805*; Q9I5D3; 3.56), S-adenosylmethionine decarboxylase proenzyme (*speH*; Q9HV35; 3.55), and proteins involved in nitrogen and amino acid metabolism, such as Glutamate synthase large chain (*gltB*; Q9HUD5; 2.64). Additional proteins in this group were MOSC domain-containing protein (*PA0529*; Q9I607; 2.90), Probable glutamine amidotransferase (*PA0531*; Q9I605; 2.70), and Cytochrome bo(3) ubiquinol oxidase subunit 1 (*cyoB*; Q9I426; 2.40).

The top 10 *P. aeruginosa* proteins most decreased in abundance in the CFS-treated group as compared to CTRL (Table 1), included multiple proteins involved in phenazine biosynthesis. Notably, Phenazine/pyocyanine biosynthesis protein PhzF (*phzF1/phzF2*; O69754) exhibited the strongest decrease in abundance, with a Log fold change of −5.17 and a Log10 (*p*-value) of 3.16. This was followed by the Probable binding protein component of ABC iron transporter PA5217 (Q9HTX3; −3.86), Phenazine biosynthesis protein PhzB1 (O69753; −3.61), Phenazine biosynthesis protein PhzD2 (*phzD2/phzD*; P0DPC1; −3.06), and Type III secretion system (T3SS) protein PcrV (G3XD49; −3.52). Other proteins with significantly decreased abundance included Guanidinobutyrase (*gbuA*; Q9I3S3; −3.46), Beta-ketoacyl-[acyl-carrier-protein] synthase III (*fabH*; Q9HYR2; −3.16), Probable pyridoxamine 5′-phosphate oxidase (*phzG1*; G3XCV4; −3.31), Pilin assembly protein (*PA3688*; Q9HXV2; −3.05), and Phenazine biosynthesis protein PhzD (*phzD2*; P0DPC1; −3.06).

Overall, the proteomic data revealed a clear differential abundance pattern in response to CFS vs. CTRL, with notable increases in proteins involved in respiration and nitrogen metabolism, and a consistent decrease in proteins related to phenazine biosynthesis and secretion systems.

When focusing on the differences between the two treatments, CFS and LA, it can be observed that the protein NirM was increased in abundance in CFS vs. CTRL (+5.53) (Figure 2a) while it was not detected in LA vs. CTRL (Figure 2b), suggesting partial activation of anaerobic respiration in the CFS group only. NirM is a small cytochrome involved in electron transfer during the nitrite reduction step of the denitrification pathway, and its increased abundance suggests a potential induction of anaerobic respiration in the CFS group only. Such modulation of NirM is more likely to reflect a redox- or respiration-associated adaptive response of *P. aeruginosa* to the specific stress conditions imposed by the CFS, rather than the activation of a defined anaerobic programme, as further addressed in Section 3.

Within the comparison between CFS and LA (Figure 2c), the protein PcrV, which was decreased in abundance under both treatments, was more markedly reduced in CFS as compared to LA (2.20). Moreover, several Type IV pilus proteins (PilG, PilM, PilN, PilY2, PilY1) were mildly reduced in CFS as compared with LA (~−0.6 to −0.9) (CFS vs. LA in Supplementary Table S1), indicating a possible modest decrease in adhesion and early biofilm formation.

#### 2.1.3. Protein Interaction Network on the Response of *P. aeruginosa* to CFS and LA

Protein–protein interaction network analysis revealed distinct clusters of proteins showing differential abundance in *P. aeruginosa* exposed to CFS and LA compared to untreated controls. Shared responses included enhanced respiratory activity and membrane remodelling.

In the CFS vs. CTRL comparison, the STRING network revealed increased abundance of proteins involved in denitrification, the citric acid cycle, and aerobic respiration, forming interconnected modules within the network. Additional clusters included proteins that showed increased abundance and were associated with urease activity, the arginine deiminase (ADI) pathway, and lipid A and polyamine metabolism. Lastly, a distinct group of proteins related to the outer cell membrane structure and remodelling was also present.

Specifically, for aerobic respiration, cyoA and cyoB, which encode subunits of cytochrome *bo* terminal oxidase, the main terminal oxidase under high-oxygen conditions, were both among the top 10 most abundant proteins, supporting the enhanced activity of this pathway (Table 1).

Consistent with the increased abundance of NirM described above, several additional nir and nor proteins within the denitrification pathway also displayed higher levels.

Regarding urease-associated proteins, UreB, UreC, UreE, and UreG showed increased abundance, with UreG, a GTPase involved in urease maturation, also among the top 10 most increased proteins (Table 1).

**Figure 3:**
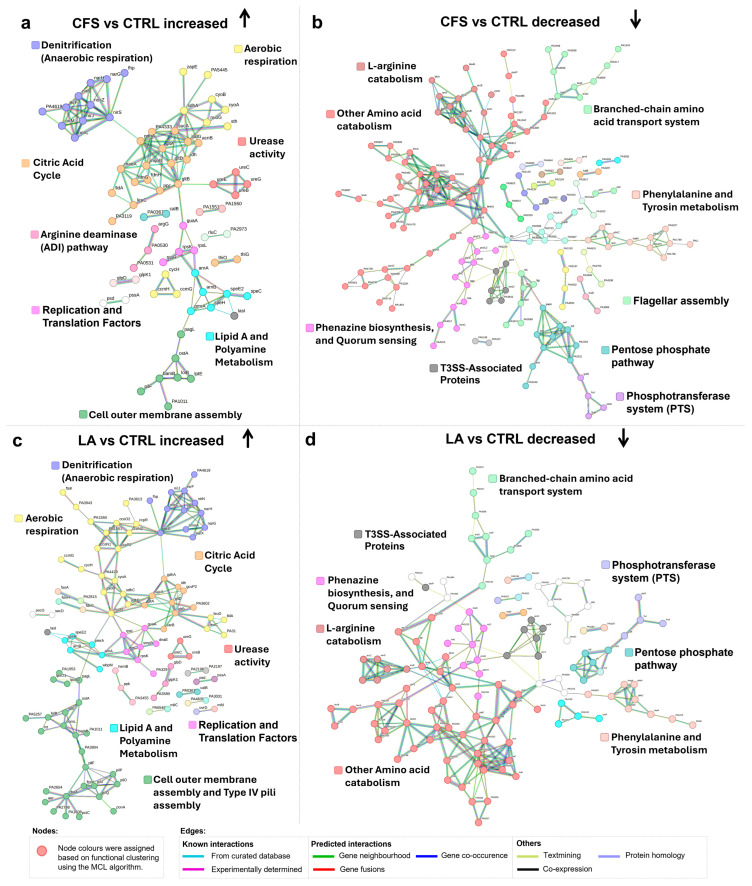
Protein Interaction Network analysis of proteins increased or decreased in abundance in *P. aeruginosa* exposed to *L. rhamnosus* cell-free supernatant (CFS) and lactic acid (LA) as compared to control (CTRL). The network highlights protein–protein interactions and functional associations. Node colours were assigned based on functional clustering using the Markov Cluster (MCL) algorithm. The edges represent functional and physical protein associations, with line colours indicating the source of interaction evidence.

Proteins involved in the citric acid cycle showed consistently increased abundance, including GltA, GltB, and GltD (citrate synthase), as well as SdhC and SdhD (succinate dehydrogenase subunits). Additionally, components of the cytochrome c oxidase complex, CcoN2 and CcoO2, together with the respiratory regulator CcpR, also showed increased abundance (See Figure 3a or Supplementary Table S1).

An additional noteworthy observation is the upregulation of the proteins ArnA and ArnB, which are involved in the modification of lipid A within lipopolysaccharides (LPS). This modification reduces the negative charge of the outer membrane, potentially enhancing resistance to environmental stressors. Concurrently, there is an upregulation of proteins associated with polyamine metabolism, such as SpeE2 and SpeH, both among the top 10 most abundant proteins (Table 1). These enzymes contribute to the biosynthesis of spermidine, a polyamine known to play a role in stress response and membrane integrity. Together, these changes suggest a coordinated adaptive mechanism aimed at reinforcing membrane stability under stress conditions.

Finally, several pil proteins (pilF in CFS vs. CTRL Figure 3a and pilF, pilJ, pilO, pilP in LA vs. CTRL Figure 3c), components of the type IV pilus system, showed increased abundance, indicating possible changes in surface structure, motility or adhesion properties.

The analysis of proteins decreased in abundance in *P. aeruginosa* treated with CFS or LA, compared to CTRL, revealed several distinct functional clusters. Key metabolic pathways and virulence-associated systems were represented among the groups showing decreased abundance. Proteins involved in amino acid catabolism, including general amino acid degradation and arginine catabolism, were significantly reduced in abundance. Components of the branched-chain amino acid transport system were also less abundant, alongside enzymes related to phenylalanine and tyrosine metabolism. A decrease in abundance was observed for proteins involved in phenazine biosynthesis, including four among the top 10 most decreased: PhzD, PhzG1, PhzB1, and PhzF1/PhzF2 (Table 1). These enzymes contribute to the production of precursors to siderophores such as pyocyanin and pyochelin, which are essential for iron acquisition and support *P. aeruginosa* survival and virulence during infection. In parallel, key quorum-sensing (QS) regulators, such as RhlR, which governs multiple virulence factors, and RsaL, a repressor involved in fine-tuning QS responses, showed decreased abundance. Interestingly, LasI, the enzyme responsible for producing the autoinducer 3-oxo-C12-HSL, was more abundant despite the overall reduction in the QS-associated proteins. We observed a marked reduction in several core components of the T3SS, a major virulence mechanism of *P. aeruginosa*. Among the most decreased was PcrV (Table 1), the needle-tip protein required for assembling the translocation complex and facilitating effector delivery into host cells. PopD, a pore-forming translocator in the host membrane, and PcrH, a dedicated chaperone that stabilises PopD and its partner PopB prior to secretion, also exhibited reduced abundance. Additionally, the effector toxin ExoS, which disrupts host cell signalling and promotes immune evasion, showed a notable decrease. This coordinated decline in T3SS components suggests a reduced virulence potential in response to treatment.

Multiple proteins involved in the pentose phosphate pathway, including Glk (glucokinase), Gap (glyceraldehyde-3-phosphate dehydrogenase), Zwf (glucose-6-phosphate dehydrogenase), Pgl (6-phosphogluconolactonase), Edd (6-phosphogluconate dehydratase), and Eda (KDPG aldolase), were reduced in abundance, indicating suppression of glycolysis (Figure 3b). Furthermore, the phosphotransferase system also showed reduced abundance, with FruI and FruK, key components of the fructose-specific phosphotransferase system, displaying lower abundance. This pattern suggests a broader reduction in glucose and fructose metabolism. Together, this leads to the conclusion that glucose and fructose may not actively be used as carbon sources under those conditions.

In the LA vs. CTRL network, a similar pattern was observed for pathways associated with proteins showing increased abundance; denitrification, citric acid cycle, aerobic respiration, urease activity, lipid A, polyamine metabolism, outer cell membrane, while pathways linked to proteins with decreased abundance mainly involved amino acid catabolism and phenazine biosynthesis., QS, T3SS-associated proteins, pentose phosphate pathway, phosphotransferase system, indicating a largely overlapping response to both treatments.

### 2.2. Analysis of the Effects of CFS and LA on the Proteome of S. aureus

#### 2.2.1. Proteomic Characterisation of *S. aureus*

To investigate the proteomic response of *S. aureus* to CFS and LA exposure, we performed a LFQ proteomic analysis comparing treated bacteria with untreated controls. Preliminary growth assessments were performed by measuring the OD_600_ of the treated cultures at the chosen time point of 6 h. The concentration of CFS used for *S. aureus* was 1:8, corresponding to sub-inhibitory concentrations, which allows measurable stress responses without extensive loss of viability (Supplementary Figure S1). A total of 1561 proteins were initially identified through proteomic analysis. After filtering out contaminants and peptides identified by site, 1513 proteins remained. Post-imputation ANOVA (*p* < 0.05, fold change > 1.5) identified 492 proteins whose abundance differed significantly in at least one of the three experimental groups (CTRL, CFS, LA) (Supplementary Table S2).

**Figure 4:**
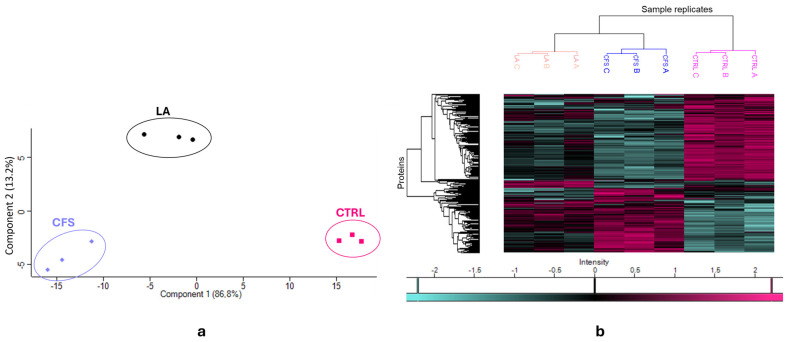
Principal components analysis (PCA) (**a**) and Heatmap (**b**) of the proteome of *S. aureus* exposed to *L. rhamnosus* cell-free supernatant (CFS) and lactic acid (LA) as compared to the untreated control (CTRL).

PCA of the *S. aureus* dataset (Figure 4a) was consistent with what was observed in Figure 1a with *P. aeruginosa.* There is a pronounced separation of the control group (CTRL) from both treatment groups (CFS and LA), particularly along principal component 1.

At the same time, CFS and LA are clustered closer to each other, indicating a higher degree of similarity between their proteomic profiles, though this time the CFS and LA groups appear to be neatly separated. This clustering pattern in the PCA of *S. aureus* is further corroborated by the heatmap analysis (Figure 4b), which also reveals a distinct proteomic profile for the CTRL group in contrast to the treated samples that appear to be more similar. However, in *S. aureus*, they are more clearly separated compared to *P. aeruginosa*; in fact, the dendrogram shows a well-defined clustering.

#### 2.2.2. Differential Proteomic Analysis of *S. aureus* Following Exposure to CFS and LA

To investigate the impact of CFS and LA treatments on the *S. aureus* proteome, volcano plots were generated using pairwise Student’s t-tests (*p* < 0.05) to identify differences in protein abundance between samples and to visualise changes in the associated pathways and biological processes.

**Figure 5:**
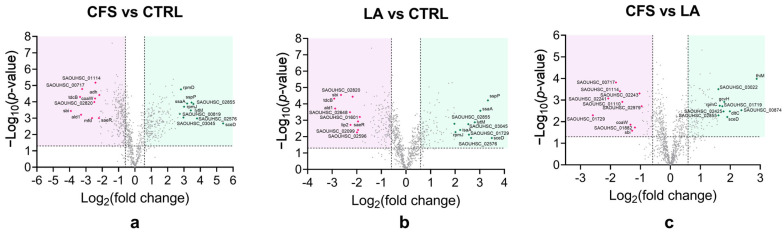
Volcano Plots of differentially expressed proteins in *S. aureus*, showing the distribution of proteins based on statistical significance (−Log10 *p*-value) and magnitude of change (Log_2_ fold change) in the proteome of *S. aureus* exposed to *L. rhamnosus* cell-free supernatant (CFS) compared to the untreated control (CTRL) (**a**), lactic acid (LA) compared to CTRL (**b**) and CFS compared to LA (**c**). Proteins above the significance threshold (*p* < 0.05) are considered differentially abundant. The top 10 proteins with increased abundance are highlighted in green, while the top 10 proteins with decreased abundance are highlighted in pink. Gene names of related proteins are indicated next to each highlighted protein.

The plots (Figure 5a–c) illustrate the distribution of proteins based on statistical significance (−Log_10_ *p*-value) and magnitude of change (Log_2_ fold change) for the three pairwise comparisons: CFS versus untreated control (CTRL), LA versus CTRL, and LA versus CFS. Proteins above the significance threshold were considered differentially expressed.

For all comparisons, the top ten proteins with the greatest increase in abundance are highlighted in green, and the ten with the greatest decrease in abundance are highlighted in pink. The proteins corresponding to the CFS vs. CTRL (Figure 5c) comparison are displayed in Table 2.

Among the top 10 most abundant *S. aureus* proteins in the CFS-treated condition compared to the untreated control (Table 2), the highest was the transglycosylase SceD (*SceD*, Q2FWF8), with a Log fold change of 5.39. This was followed by Staphopain A (*sspP*; Q2G2R8) with a Log fold change of 3.46, a cysteine protease associated with virulence, Glycyl-glycine endopeptidase LytM (O33599; 3.41), which may contribute to cell wall remodelling. Other highly increased-abundance proteins were Staphylococcal secretory antigen SsaA (Q2FV55; 3.19), and two ribosomal proteins: 50S ribosomal protein L36 (*rpmJ*; Q2FW29; 3.00), 50S ribosomal protein L30 (*rpmD;* 2.81). Additional proteins with increased abundance included several uncharacterised proteins.

On the other hand, among the top 10 least abundant *S. aureus* proteins in the CFS-treated condition compared to the untreated control (Table 2), the highest was Immunoglobulin-binding protein Sbi (*Sbi*, Q2FVK5), with a Log fold change of –3.92. Other proteins with markedly decreased abundance included L-threonine dehydratase catabolic TdcB (Q2FYJ3: −3.36), Alanine dehydrogenase 1 (*ald1*; Q2FYJ2; −3.30), Transcription-repair-coupling factor Mfd (Q2G0R8; 2.62), and Type II pantothenate kinase (*coaW*; Q2FWC7; −2.44). Other proteins with decreased abundance included Response regulator SaeR (Q2G2G2; −2.21), suggesting potential repression of stress and virulence-associated pathways, Alcohol dehydrogenase (*adh;* Q2G0G1; −2.18), and several uncharacterised proteins.

Comparative proteomic analysis between CFS and LA treatments revealed distinct differences in *S. aureus* protein abundance, as shown in Figure 5c. Metabolic processes included enzymes involved in vitamin and amino acid metabolism, such as ThiM (Q2FWG2; +2.88), GcvH (Q2FZZ8; +1.80), and Ald2 (Q2FXL7; +0.59), which were significantly abundant in CFS compared to LA (CFS vs. LA in Supplementary Table S2). Cell wall proteins were also differentially affected. CFS treatment led to an increase in the abundance of proteins involved in peptidoglycan turnover and modification compared to LA, including SceD (Q2FWF8; 1.90) and DltC (Q2FZW4; +1.98) (CFS vs. LA in Supplementary Table S2). Stress response proteins were modestly overexpressed under CFS vs. LA comparison, with chaperones GroS (Q2FWN3; +1.29), GrpE (Q2FXZ1; +1.08), redox-related proteins TrxA (Q2FZD2; +1.25), and superoxide dismutases SodM (Q2G261; +1.39) and SodA (P0A0J3; +1.34) showing decreased levels (ranging from +1.08 to +1.39) (CFS vs. LA in Supplementary Table S2). Within the translational machinery, CFS exposure as compared to LA resulted in upregulation of ribosomal proteins from both large and small subunits, RpmC (Q2FW14; +1.65), RplX (Q2FW17; +1.12), RpsP (Q2FZ45; +1.41), as well as transcription-associated RpoE (Q2FWD0; −1.35), with fold increases between +1.12 and +1.65 (CFS vs. LA in Supplementary Table S2). Finally, changes were observed in virulence-associated proteins. Protein A (Spa; P02976; +1.11) was modestly increased under CFS (CFS vs. LA in Supplementary Table S2).

#### 2.2.3. Protein Interaction Network on the Response of *S. aureus* to CFS and LA

**Figure 6:**
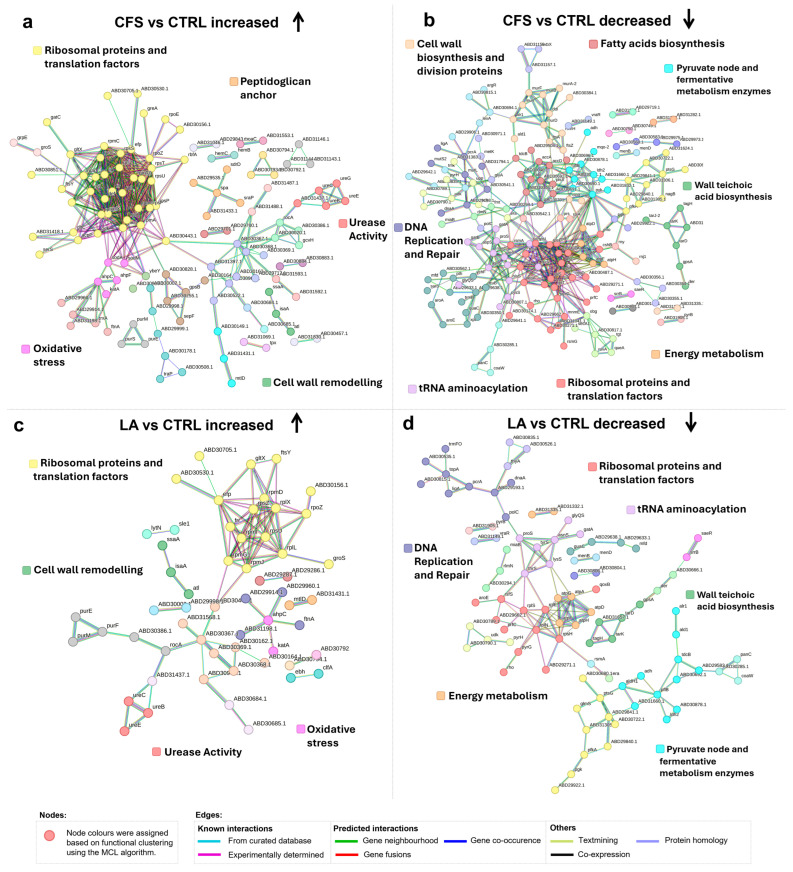
Protein Interaction Network analysis of proteins increased or decreased in abundance in *S. aureus* exposed to *L. rhamnosus* cell-free supernatant (CFS) and lactic acid (LA) as compared to control (CTRL). The network highlights protein–protein interactions and functional associations. Node colours were assigned based on functional clustering using the Markov Cluster (MCL) algorithm. The edges represent functional and physical protein associations, with line colours indicating the source of interaction evidence.

Protein–protein interaction network analysis revealed distinct clusters of proteins increased in abundance in *S. aureus* exposed to CFS and LA compared to untreated controls (Figure 6). A marked increase was observed in proteins associated with translation, particularly ribosomal components. Among these, several 50S ribosomal proteins (RpmJ, RpmD, RplI, RplK, RplL, RplQ, RplX) and 30S ribosomal proteins (RpsF, RpsP, RpsS, RpsT, RpsU, RpsZ) displayed increased abundance. Translation-associated factors, including the initiation and elongation proteins Efp, Tsf and InfA, displayed increased abundance, together with auxiliary proteins involved in protein folding and processing, such as Tig and Map. In addition to the ribosomal cluster, several oxidative stress–response proteins (SodA, SodM, KatA, AhpC and AhpF) were also more abundant. Urease-related proteins, including UreB, UreC, UreE and UreG, likewise exhibited higher abundance. A rise in abundance was further observed for proteins associated with peptidoglycan turnover and cell wall remodelling, with autolysins IsaA and Atl among the most represented. Finally, surface-associated, peptidoglycan-anchored proteins such as Spa, SraP and SdrD were also more abundant under these conditions.

In contrast, several protein clusters were reduced in abundance. Within the translational machinery, decreased abundance was observed in a number of ribosomal proteins, including 50S subunits (RplD, RplE, RplN, RplS, RplT) and 30S proteins (RpsC, RpsE, RpsI, RpsR). Key elongation factors (FusA, LepA), together with proteins involved in transcription–translation coupling and RNA regulation (CshB, NusA, Rho, TrmD), were also reduced.

A clear suppression of DNA replication proteins was detected, including DNA polymerase III catalytic subunit (PolC) and the replication initiator DnaA. Proteins involved in DNA repair and nucleotide metabolism, such as MutL, MutS2, and enzymes linked to pyrimidine metabolism, were similarly reduced in abundance. Energy metabolism was negatively affected, as evidenced by the decreased abundance of multiple ATP synthase subunits (AtpA, \, AtpF, AtpG, AtpH). Decreased abundance also extended to wall teichoic acid biosynthesis proteins, including TarD, TagH, and TarK. Finally, proteins involved in metabolic remodelling, such as Adh, Ald1, and TdcB, showed reduced expression.

In addition, several proteins related to fatty acid biosynthesis were differentially expressed. Acetyl-CoA carboxylase subunits (AccA, AccD), enoyl-ACP reductase (FabI), and the fatty acid/phospholipid synthesis protein PlsX were consistently reduced in abundance, suggesting an impairment of membrane lipid metabolism under CFS and LA treatment.

## 3. Discussion

In this study, we analysed the proteomic responses of *P. aeruginosa* and *S. aureus* to LA and CFS from *L. rhamnosus*. Using LFQ proteomics, we identified interesting strain-specific changes in proteins linked to acidic stress, metabolism, and virulence that are discussed below. To ensure that the proteomic analysis captured biologically meaningful adaptive responses, we performed preliminary growth assessments for both *P. aeruginosa* and *S. aureus* under the selected treatment conditions. As shown in Supplementary Figure S1, the concentrations used correspond to sub-inhibitory levels that maintain sufficient bacterial growth while still inducing a measurable physiological response, consistent with established LFQ proteomics workflows [40,42,43].

### 3.1. Proteomic Response of P. aeruginosa to CFS and LA

#### 3.1.1. Upregulation of Denitrification, Aerobic Respiration and Citric Acid Cycle

One interesting finding in our analysis was the upregulation of the denitrification pathways in *P. aeruginosa* upon exposure to both LA and CFS. The denitrification is a form of anaerobic respiration that allows the bacterium to use nitrate and nitrite as alternative electron acceptors under low-oxygen conditions, a process regulated by the transcription factors Anr and Dnr, which are activated in response to hypoxia and nitric oxide and promote the expression of nitrate and nitrite reductases [44,45].

Although the bacterial cultures were not likely exposed to low oxygen, the activation of denitrification could serve an alternative physiological role, such as pH regulation, since the process can result in ammonia production, which helps counter acidic stress [45].

Interestingly, we also observed concurrent upregulation of aerobic terminal oxidases and of the citric acid cycle, suggesting that *P. aeruginosa* is not exclusively switching to anaerobic respiration but rather activating multiple energy-generating pathways simultaneously. Acidity and hypoxia often co-occur in chronic infection environments such as the cystic fibrosis lung, and their regulatory responses may be tightly linked, meaning that sensing one condition may trigger responses to both [46].

This dual activation aligns with prior observations showing that *P. aeruginosa* can co-regulate aerobic and anaerobic respiratory pathways to optimise survival in complex, spatially heterogeneous environments [45].

#### 3.1.2. pH Neutralisation via Urease and Arginine Deiminase in *P. aeruginosa*

The observed upregulation of the urease and ADI pathways likely reflects a bacterial strategy to counteract cytoplasmic acidification by producing ammonia, which neutralises excess protons and helps maintain intracellular pH homeostasis. The ADI pathway is a well-characterised acid resistance mechanism across diverse bacterial taxa [47]. In *Streptococcus pyogenes*, activation of the ADI pathway was described to enhance survival under acidic conditions by buffering the cytoplasm and generating ATP to support protective systems such as the F_1_F_0_-ATPase [48]. Similarly, in *Staphylococcus epidermidis* and *S. aureus*, the ADI-derived ammonia was also described as a survival strategy to acid [49,50].

Although the ADI pathway is well characterised and predominantly employed as an acid resistance mechanism in Gram-positive bacteria, particularly streptococci and staphylococci [47,49,50], its role in Gram-negative species is less conserved and generally less prominent. Nonetheless, isolated reports have described ADI-associated genes or activity in some Gram-negative bacteria, indicating that the pathway may be present in a limited or condition-dependent manner [51,52].

Urease pathway contributes to the cytoplasmic buffering capacity, catalyses the hydrolysis of urea to ammonia and carbamate; the latter spontaneously decomposes to yield additional ammonia and carbonic acid [47,53]. The increased-abundance UreB and UreC are structural subunits of the urease machinery, along with UreE and UreG, that are metallo-chaperones essential for urease activation by facilitating the incorporation of nickel into the enzyme complex and activation proteins [54].

Urease-mediated alkalization is a well-described survival strategy among gastrointestinal bacteria facing extreme acid stress. For instance, *Laribacter hongkongensis* employs this mechanism to withstand acidic environments [55]. Similarly, *Helicobacter pylori* relies on high urease activity as a key adaptation, enabling survival in the highly acidic gastric milieu by neutralising stomach acid [56].

#### 3.1.3. Outer Cell Membrane Protection from Acidity

Another well-described consequence of acid stress is the modification of the cell membrane [47], in particular, the remodelling of lipid A, the negatively charged hydrophobic anchor of LPS in the outer membrane of Gram-negative bacteria. Under mildly acidic conditions, *P. aeruginosa* activates genes of the Arn operon, leading to the addition of 4-amino-4-deoxy-L-arabinose (Ara4N) to lipid A [57]. This modification neutralises the negative charge on phosphate groups of lipid A, which would otherwise attract cationic antimicrobial peptides (CAMPs) like polymyxins and other host immune protection mechanisms.

In parallel, *P. aeruginosa* upregulates polyamine biosynthesis genes (spe), which produce putrescine and spermidine, small positively charged molecules that help stabilise bacterial surfaces. Polyamines interact with negatively charged membrane components like LPS and phospholipids to form non-covalent electrostatic networks that reduce membrane fluidity and permeability, also reducing its sensitivity to membrane-active antibiotics [58,59].

The upregulation of type IV pilus proteins in *P. aeruginosa* under mildly acidic conditions likely reflects an adaptive response to enhance adhesion and persistence. In other species, such as *Escherichia coli* and *Lactobacillus rhamnosus*, acid stress has been shown to increase pilus gene expression, promoting stronger attachment to epithelial or mucosal surfaces as a survival strategy [60,61]. While direct evidence in *P. aeruginosa* is limited, its type IV pili are known to be tightly regulated by environmental cues and play key roles in motility, colonisation, and virulence [62,63].

#### 3.1.4. Virulence-Associated Pathways: Phenazine Biosynthesis, Quorum-Sensing and T3SS

Several key virulence pathways or virulence-related pathways, including QS, phenazine biosynthesis, and the T3SS, consistently exhibited reduced abundance in *P. aeruginosa* following treatment with both CFS and LA.

The decrease in abundance of key QS regulators, such as RhlR and RsaL, suggests that QS is suppressed in our experimental conditions; nevertheless, LasI resulted increased in abundance. This appears contradictory, given its typical activation by upstream QS regulators. However, previous studies have demonstrated that environmental conditions can disrupt the classical QS hierarchy, leading to uncoupling between autoinducer production and downstream regulatory activity [64]. Given the simultaneous reduction in abundance of RhlR, RsaL, and multiple virulence-associated proteins, the upregulation of LasI likely reflects a stress-related or compensatory response rather than effective QS activation.

Recent studies support the idea that LA specifically suppresses QS-regulated virulence mechanisms in *P. aeruginosa*. For example, Kiymaci and colleagues showed that LA produced by the potential probiotic *Pediococcus acidilactici* inhibited the production of QS signalling molecules and associated virulence traits, including pyocyanin, protease, and biofilm production, all of which are controlled by QS systems [65]. Similarly, Sonbol and colleagues found that weak acids, particularly LA, significantly downregulated QS genes such as *lasI*, *lasR*, *rhlI*, and *rhlR*, resulting in marked reductions in multiple virulence factors [66].

This suppression extends to phenazine biosynthesis; phenazines like pyocyanin are downregulated when QS is inhibited, as demonstrated in studies showing that QS disruption leads to reduced *phz* operon expression. CFS has already been shown to have anti-virulence properties against cystic fibrosis clinical isolates of *P. aeruginosa* and to be able to decrease the production of pyocyanin in the pathogen [34].

Finally, although no direct studies have investigated the downregulation of T3SS components in *P. aeruginosa* following exposure to either CFS or LA, some related works have reported the regulation of other secretion systems in *P. aeruginosa* [67] as well as T3SS regulation in other pathogens by high cell density [68].

In *P. aeruginosa* PA14, the H3-type VI secretion system (H3-T6SS), another contact-dependent secretion system, has been shown to be regulated by OxyR and OmpR, global regulators of oxidative and acid stress, demonstrating a mechanistic link between acid stress signalling and secretion system control [67]. Moreover, recent work in *Yersinia enterocolitica* has shown that high cell density alone can actively repress T3SS expression through regulatory RNAs and the transcription factor VirF, indicating that bacteria may downregulate energy-intensive virulence systems in response to environmental cues unrelated to host contact [68]. Although this repression is not directly linked to acid stress, it supports the broader idea that virulence systems like T3SS are tightly regulated in response to non-host, non-ideal conditions.

#### 3.1.5. Reduced Amino Acid Catabolism as Part of Carbon Source-Driven Metabolic Shifts

The decreased amino acid catabolism observed in *P. aeruginosa* upon exposure to CFS and LA is consistent with the metabolic strategy known as carbon catabolite repression (CCR), specifically the “reverse diauxie” phenomenon. In *P. aeruginosa*, unlike classical model organisms such as *E. coli*, organic acids like lactate serve as the preferred carbon sources over amino acids or glucose, leading to the suppression of less favourable metabolic pathways [69,70]. A prominent feature of this response is the upregulation of LldA, the L-lactate dehydrogenase responsible for converting LA to pyruvate, facilitating its direct entry into central metabolism [71], which was also found to be increased in abundance in this work. Moreover, as support of this metabolic preference, we observed an increase in abundance of several key components of the citric acid cycle, and a decrease in abundance of glucose metabolism, pentose phosphate pathway and phosphotransferase system, that would align with the metabolic shift described in the CCR phenomenon.

In conclusion, *P. aeruginosa* exposure to both the CFS and LA triggered a clear metabolic adaptation characterised by the activation of lactate catabolism and respiratory pathways, coupled with a marked repression of amino acid and sugar utilisation. This shift is consistent with the reverse diauxie phenomenon, whereby *P. aeruginosa* optimises energy efficiency by prioritising preferred carbon sources derived from CFS or LA, while reducing the use of energetically costly alternative catabolic pathways.

Above all, the reverse diauxie phenomenon may not contradict with the antimicrobial potential of CFS. In this study, both CFS and LA were used at sub-inhibitory concentrations. Previous research clearly demonstrates that at therapeutically relevant concentrations, LA exhibits strong antimicrobial activity against *P. aeruginosa*, effectively inhibiting bacterial growth and biofilm formation in both clinical isolates from cystic fibrosis patients and chronic infected wounds [18,33,34]. Notably, sub-inhibitory concentrations, while not lethal, can still weaken pathogenic traits by downregulating virulence pathways as previously discussed, and this dual mechanism, potentially sensitising the bacteria to subsequent antimicrobial treatment or immune clearance and reducing their pathogenic potential even before reaching bactericidal levels.

#### 3.1.6. CFS and LA Comparison: *P. aeruginosa*

This proteomic comparison between *P. aeruginosa* exposed to CFS and LA revealed little distinct physiological response, with few proteins showing pronounced differential abundance. The overall patterns suggest that the treatments are largely similar, but subtle differences are present. Specifically, while both treatments impact similar pathways to some extent, CFS appears to exert a more suppressive effect on secretion systems, whereas LA induces a stronger oxidative stress response and slightly enhances surface-adhesion-related proteins.

One of the most notable differences was observed for NirM (Log fold change of 5.53 in CFS, not present in LA SSDAs), a small c-type cytochrome involved in electron transfer to nitrite reductase during denitrification. Although other components of the denitrification pathway were not differentially expressed, the marked upregulation of NirM may reflect an early or partial activation of anaerobic respiration. Notably, NirM plays a crucial functional role in denitrification: it acts as a primary electron donor to NirS, the cytochrome cd_1_-type nitrite reductase, and is indispensable for enabling its activity [72]. Therefore, the upregulation of NirM, despite the absence of other Nir proteins, may still indicate a physiologically meaningful adjustment in the denitrification machinery.

PcrV, a structural protein of the type III secretion system (T3SS), was reduced in abundance under both conditions, but more strongly in CFS (CFS vs. CTRL fold change −3.52) compared to LA (LA vs. CTRL fold change −1.72). This suggests a general suppression of T3SS expression in both environments, with CFS exerting a stronger repressive effect, possibly through signalling molecules that interfere with virulence regulation pathways.

Several proteins involved in Type IV pilus assembly (*pilG*, *pilM*, *pilN*, *pilY2*) were decreased in abundance in CFS as compared to LA (fold changes~−0.6–−0.9) (Supplementary Figure S2). Type IV pili are critical for twitching motility, biofilm initiation, and surface adherence in *P. aeruginosa* [73]. Importantly, studies have shown that disruption or downregulation of key pilus components leads to significantly impaired adhesion and reduced biofilm formation capacity [74]. Specifically, the pilin receptor-binding domain has been shown to function directly as an adhesin, and loss of this function prevents bacterial attachment to surfaces like stainless steel and epithelial cells. Moreover, PilY1, which was among the proteins with decreased abundance in CFS vs. LA in this study, plays a dual role as an adhesin and a regulator of pilus dynamics. Its absence or suppression has been associated with a significant reduction in surface piliation and bacterial adherence [75]. Therefore, the observed reduction in Type IV pilus proteins in response to CFS likely reflects a broader impairment in pilus-mediated attachment mechanisms. Taken together, this suggests that CFS from *L. rhamnosus* may more effectively reduce *P. aeruginosa* adherence than LA alone, potentially through specific inhibitory components that interfere with the pilus assembly machinery. This interference could contribute to a decreased ability of the bacteria to initiate biofilm formation and colonise surfaces. This hypothesis is strongly supported by our recent findings showing an impairment of early-phase biofilm formation in *P. aeruginosa* in an air-liquid-interface lung infection model along with CFS treatment [35]. However, due to the modest magnitude of these differences, their biological relevance here remains to be further confirmed.

### 3.2. Proteomic Response of S. aureus to CFS and LA

#### 3.2.1. pH Neutralisation via Urease in *S. aureus*

As already discussed for *P. aeruginosa* in Section 3.1.2, the urease pathway contributes to cytoplasmic buffering by catalysing the hydrolysis of urea into ammonia and carbon dioxide, thereby raising intracellular pH and counteracting acid stress [47,53]. In the *S. aureus* dataset, we observed a similar upregulation of the urease pathway protein as discussed with *P. aeruginosa*, with UreB and UreC (structural subunits) along with UreE and UreG (nickel-binding and activation proteins) [54]. This aligns with previous reports where acid shock led to strong urease induction in *S. aureus*, enabling direct pH neutralisation [49,76].

Overall, the urease pathway in *S. aureus* is a well-characterised acid resistance mechanism whose coordinated activation has been repeatedly linked to enhanced survival in acidic environments and host niches, underscoring its importance as a robust and evolutionarily conserved component of the organism’s stress adaptation and virulence repertoire [49].

#### 3.2.2. Oxidative Stress Response Under Acidic Conditions

Oxidative stress is indirectly triggered by acid stress through the accumulation of reactive oxygen species (ROS) and plays a critical role in bacterial survival under hostile conditions.

Following treatment of *S. aureus* cultures with CFS and LA, significant upregulation of the *S. aureus* ROS detoxification systems was observed, such as superoxide dismutase (SOD) (sodA and sodM), catalase (katA), alkyl hydroperoxide reductase (aphC and ahpF). These results align well with prior observations from Bore and colleagues, who reported upregulation of sodA, katA, ahpC, and ahpF under abrupt acid-shock conditions, highlighting a coordinated oxidative-stress response linked to acid adaptation [49,76].

In *S. aureus*, the superoxide dismutases SodA and SodM play important roles in protecting the cell from the combined effects of acid and oxidative stress. Acid stress has been shown to induce the expression of Mn-dependent superoxide dismutase (SodA) in *S. aureus*, helping the bacteria survive in low pH environments by detoxifying ROS generated under these conditions [77].

In addition to SodA, most commonly studied in *S. aureus*, the bacterium also expresses SodM, a unique isoform capable of functioning with either manganese or iron. This versatility likely provides additional protection when manganese is limited, such as during acid stress or host-imposed nutritional immunity [78].

The elevated expression of KatA and the AhpC/AhpF system underscores their importance in neutralising reactive oxygen species generated either directly or indirectly by acid stress. These components function in a compensatory manner, whereby KatA handles high H_2_O_2_ levels and AhpC/AhpF detoxifies broader ROS, with regulatory control via PerR [79,80].

To conclude, the observed oxidative stress response following LA and CFS treatment is consistent with previous findings on acid stress adaptation in *S. aureus*.

#### 3.2.3. Ribosomal Remodelling and Reduced DNA-Related Protein Abundance Suggest a Bacteriostatic-like Response

The alteration of ribosomal proteins, translational proteins and initiation factors, observed in the CFS and LA treatments, may be an adaptive mechanism aimed at maintaining protein homeostasis and managing damage control mechanisms, in order to prioritise survival over growth.

A similar trend has been reported in transcriptomic analyses by Peng and colleagues, where *S. aureus* exhibited enhanced expression of stress-related transcripts alongside repression of growth-associated pathways in response to probiotic CFS [81]. Their study demonstrated a significant downregulation of translation-related genes, particularly ribosomal proteins and elongation factors, alongside suppression of DNA replication and nucleotide metabolism pathways [81]. Interestingly, the protein-level response appeared more nuanced, as certain ribosomal proteins (e.g., RpmJ, RpmD) were among the most increased in abundance, even though several elongation factors showed reduced abundance. This may reflect post-transcriptional adjustments or differential protein turnover under stress.

In our data, the decrease in abundance of ATP synthase subunits aligns with findings from Mao and colleagues, where *S. aureus* under probiotic CFS exhibited suppressed energy metabolism and reduced nucleotide biosynthesis [82]. Peng and colleagues similarly reported downregulation of ATP production pathways. These results combined point to an energy-consuming shift towards cellular maintenance rather than proliferation.

Taken together, the comparison between proteomic, transcriptomic, and metabolomic evidence suggests that CFS and LA may impose a coordinated bacteriostatic state in *S. aureus*, characterised by suppressed replication and metabolic slowdown while maintaining essential stress-responsive translation. This profile is consistent with the interpretation that probiotic-derived products induce a survival-oriented phenotype in *S. aureus*.

#### 3.2.4. Cell Wall Remodelling and Virulence Factors

The proteomic analysis revealed a relevant upregulation of proteins involved in peptidoglycan turnover and cell wall remodelling. Among the most prominent were autolysins such as Atl, IsaA, LytM, and SceD [83,84]. Although these proteins are canonically classified as secreted enzymes that localise to the extracellular space to cleave peptidoglycan, we detected them in the intracellular proteome, suggesting altered localisation. A likely explanation for this intracellular accumulation is that inhibition of wall teichoic acid (WTA) biosynthesis, also observed through the reduction in several proteins, among which, TarD, TagH, and TarK, disrupts autolysin export. Previous work showed that exposure to Targocil, a WTA synthesis inhibitor, leads to accumulation of full-length Atl within the membrane due to impaired export and processing. This results in reduced surface activity of autolysins despite unaltered gene expression, indicating that proper WTA synthesis is required for autolysin trafficking [85].

While our proteomic detection of autolysins in the intracellular fraction is consistent with WTA suppression–induced trafficking defects, we cannot exclude methodological factors contributing to this apparent localisation. Nevertheless, additional regulatory mechanisms may also be at play. For example, overexpression of the small RNA SprX, which is activated under stress, has been shown to upregulate transcription of isaA, atl, and lytM via the WalKR system, while not necessarily promoting secretion, leading to intracellular accumulation of autolysin transcripts and proteins [86].

Interestingly, in parallel with WTA suppression, we observed the upregulation of several leucine-proline-X-threonine-glycine (LPXTG)-motif surface proteins, including Spa, SraP, and SdrD, which are covalently anchored to the peptidoglycan by the transpeptidase sortase A [87]. These proteins do not require wall teichoic acids for their localisation or anchoring, which could render them functionally advantageous under conditions of impaired WTA biosynthesis. Complementing this shift in surface protein expression, we observed strong upregulation of the cysteine protease SspP (Staphopain A), which plays a key role in immune evasion by degrading chemokines and antimicrobial peptides, thereby impairing neutrophil recruitment and bacterial clearance [88].

Together, these findings suggest that under CFS and LA-induced stress, *S. aureus* initiates a coordinated envelope remodelling response: autolysins are transcriptionally activated but accumulate intracellularly due to WTA suppression, while peptidoglycan-anchored immune modulators and proteolytic virulence factors like SspP are increased in abundance. This reflects a flexible and highly regulated adaptation, balancing metabolic cost, envelope stress, and immune evasion.

Proteomic analysis revealed a strong decreasing of the SaeR regulon following exposure to CFS and LA. The SaeRS two-component system is a key positive regulator of multiple virulence factors [89], including α-hemolysin (*hla*), PVL toxin genes (*lukS/F-PV*), and immune evasion proteins (*sbi*, *efb*), by directly binding a conserved promoter motif upstream of these genes [90].

Interestingly, the results are consistent with transcriptomic evidence showing that acidic environments repress this regulatory system. Under acid shock at pH 4.5, both *saeS* and *saeR* were significantly downregulated along with other virulence-associated genes [76]. Similar findings were reported by Weinrick and colleagues, where exposure to mild acid likewise resulted in reduced *saeR/S* transcript levels [91].

Among the SaeR-regulated targets affected in our dataset was *sbi* (−3.92) (Table 2), which encodes the *staphylococcal* binder of immunoglobulin. *Sbi* interferes with complement activation by binding to complement factors H and C3, and leading to opsonisation evasion [92]. Downregulation of *sbi* under acidic conditions could therefore compromise *S. aureus*’ ability to evade opsonophagocytic killing, representing a potential functional consequence of SaeR repression.

Taken together, these results indicate that exposure to CFS and LA triggers a cell envelope remodelling in *S. aureus*. The suppression of WTA biosynthesis appears to impair autolysin export, leading to their intracellular accumulation, while possibly favouring the expression of LPXTG-anchored surface proteins.

In parallel, the modulation of virulence factors, including the upregulation of the cysteine protease SspP and the repression of *sbi*, suggests a broader adaptive response aimed at balancing cell envelope maintenance with immune evasion. Collectively, these findings support the idea that under acidic stress, *S. aureus* may adopt a less invasive, surface-anchored phenotype that promotes persistence and survival, enhancing its ability to survive on host surfaces, even when its capacity for tissue invasion is dormant.

#### 3.2.5. Reduced Fatty Acids Biosynthesis

Several proteins involved in fatty acid biosynthesis in *S. aureus*, including acetyl-CoA carboxylase subunits (accA, accD), enoyl-ACP reductase (FabI), and the fatty acid/phospholipid synthesis protein PlsX, were found to be reduced in abundance after treatment. The repression of these enzymes suggests a disruption of the type II fatty acid synthesis (FASII) pathway, a central process for bacterial membrane lipid production and cell viability. Targeting fatty acid biosynthesis is a well-established antibacterial strategy: for example, the frontline tuberculosis drug isoniazid exerts its bactericidal activity by inhibiting the enoyl-ACP reductase InhA, a homologue of FabI, thereby blocking mycolic acid and fatty acid synthesis [93]. Similarly, triclosan, a broad-spectrum antimicrobial, inhibits FabI in *S. aureus*, resulting in impaired fatty acid elongation and membrane synthesis [93]. The observation of decreased fatty acid biosynthetic protein abundance in the present work, therefore, suggests that interference with lipid metabolism may contribute to the antibacterial mechanism of action of CFS.

#### 3.2.6. Metabolic Remodelling

The decreased abundance of key metabolic enzymes such as alcohol dehydrogenase (Adh, 2.18), alanine dehydrogenase (Ald1, 3,30), and L-threonine dehydratase (TdcB, 3.36), which are among the least abundant proteins (Table 2), following exposure to LA and CFS was observed.

The decrease in abundance of alanine dehydrogenase (Ald1), which converts alanine to pyruvate while producing NADH, may at first appear contradictory to the idea of metabolic reprogramming toward survival, given its role in energy generation. However, in the context of high external lactate and potential redox sufficiency, repression of Ald1 is likely a protective response to avoid further NADH accumulation. Previous studies have shown that *S. aureus* employs the redox-sensitive regulator Rex to suppress NADH-generating enzymes under redox-balanced conditions [94]. Furthermore, the availability of external carbon sources such as lactate may activate carbon catabolite repression via CcpA, further downregulating amino acid catabolic pathways like alanine utilisation [95]. Thus, Ald1 repression may serve to prevent redox imbalance rather than to limit energy production per se.

In nutrient-limited or redox-challenged environments, *S. aureus* has been shown to shift from energy-intensive pathways toward more conservative metabolic strategies. For instance, when preferred carbon sources are scarce or redox homeostasis is disrupted, *S. aureus* represses ethanol production by downregulating alcohol dehydrogenase, thereby conserving acetyl-CoA for acetate production, a more ATP-efficient route [96]. The additional repression of *idh2*, encoding NADP-dependent isocitrate dehydrogenase, supports a broader metabolic shift away from oxidative TCA cycle activity toward reduced carbon flux, consistent with conditions that deprioritise energy-intensive respiration [97].

Interestingly, the decrease in abundance of *coaW* further supports this metabolic shift. CoaW is involved in the biosynthesis of coenzyme A (CoA), which is essential for numerous metabolic pathways, particularly those linked to fatty acid metabolism, TCA cycle activity, and redox homeostasis. *S. aureus* lacks glutathione and instead relies on a CoA/CoA disulfide system to manage oxidative stress [98]. Its suppression suggests a further decrease in CoA-dependent metabolic activity, reinforcing the broader trend toward metabolic conservation.

The repression of Adh observed here is consistent with this metabolic shift. Similarly, alanine dehydrogenase (Ald1), which converts alanine to pyruvate while generating NADH, may be downregulated to prevent excess NADH accumulation and protect redox balance under stress [99].

While this study did not focus primarily on biofilm formation, it is worth noting that alcohol dehydrogenases have also been implicated in the regulation of bacterial surface behaviours. In *S. aureus*, ADH expression has been correlated both positively and negatively with biofilm formation capacity, suggesting it might play a regulatory role in biofilm formation and motility [100].

The decrease in abundance of threonine dehydratase (TdcB), an enzyme involved in converting threonine to 2-ketobutyrate and ammonia, further supports the idea of restricted amino acid catabolism. Under stress, especially in low-glucose or anaerobic environments, *S. aureus* limits degradation of amino acids like threonine to preserve nitrogen and reduce metabolic burden [95].

Together, the reduction in abundance of Adh, Ald1, and TdcB suggests that *S. aureus* responds to LA exposure by reprogramming its metabolism away from NADH-generating and amino acid-catabolizing pathways, favouring redox balance and metabolic conservation under conditions that mimic host-associated or environmentally stressful niches.

#### 3.2.7. CFS and LA Comparison: *S. aureus*

Our comparative proteomic analysis revealed that the protein expression profiles of *S. aureus* treated with CFS and LA are indeed similar to those already observed for *P. aeruginosa*, but contain some interesting relevant differences as detailed below. An additional layer of distinction between CFS and LA emerges when considering the protein composition of the CFS itself. In a previous proteomic analysis [18], we identified 37 high-confidence proteins in the CFS, notably including several cell wall hydrolases, enzymes that play critical roles in peptidoglycan remodelling, separation during cell division, and autolysis that could be particularly relevant against Gram-positive organisms like *S. aureus* [101,102]. Importantly, these probiotic secreted proteins were absent in LA, further supporting the idea that CFS exerts a unique and complex form of stress beyond simple acidification. While both treatments influence bacterial physiology, the presence of unique proteins and their elevated levels in the CFS condition suggest a more complex mode of action.

- **Metabolic Remodelling**

While both CFS and LA triggered a decrease in several energy- and NADH-generating enzymes, including Ald1, Adh, and TdcB, consistent with a shift toward redox balance and metabolic conservation, CFS also uniquely induced specific metabolic enzymes, pointing to a more complex reprogramming. In particular, the upregulation of ThiM (+2.88) and GcvH (+1.80) under CFS suggests that *S. aureus* does not simply repress central metabolism but may actively adjust cofactor biosynthesis and amino acid flux to support survival under more multifaceted stress.

ThiM, a key enzyme in thiamine (vitamin B1) biosynthesis, provides thiamine phosphate, a critical cofactor for TPP-dependent enzymes involved in energy metabolism and redox homeostasis [103]. Its upregulation under CFS may help the cell keep producing enough thiamine to support essential enzyme activities during stress, especially when the demand for metabolic and redox balance is increased.

Similarly, GcvH (Supplementary Figure S2), a component of the glycine cleavage system, helps the cell break down glycine to generate molecules needed for DNA synthesis and antioxidant defences, such as one-carbon units and NAD(P)H. These products are especially important under oxidative or nutrient stress, when the cell must repair damage and control reactive oxygen species. In *S. aureus*, GcvH has also been implicated in lipoate metabolism and nutrient stress responses, underscoring its adaptive metabolic role under challenging conditions [104].

It is plausible that specific components of the CFS, such as redox-active compounds, extracellular enzymes, or nutrient signals, induce this response by creating a stress environment that mimics host conditions. In this context, the upregulation of ThiM and GcvH may help *S. aureus* produce vital cofactors, manage redox balance, and maintain metabolic activity under pressure.

- **Autolysins and Cell Wall-Related Proteins**

The elevated abundance of SceD (+1.90) and dltC (+1.98) (Supplementary Figure S2) proteins in CFS-treated samples are particularly noteworthy. As previously discussed, autolysins such as SceD are normally secreted to remodel peptidoglycan, but their intracellular accumulation can signal defective trafficking due to perturbed WTA biosynthesis. The fact that SceD is more abundant under CFS treatment than LA therefore suggests that CFS imposes stronger stress on protein export and cell envelope integrity. This mirrors observations under conditions like bicarbonate depletion, where SceD upregulation accompanies environmental stress responses [105]. The accumulation under CFS may thus represent a priming toward autolysis or maladapted remodelling, highlighting a fundamental difference from LA, where such disruption is less evident.

The dltC gene is part of the *dlt* operon, which mediates D-alanylation of WTAs. This modification reduces the net negative surface charge of *S. aureus*, thereby decreasing the binding affinity of CAMPs, an essential component of host innate immunity. Multiple studies have shown that loss of dlt function increases susceptibility to CAMPs and reduces survival in vivo [106,107].

In our dataset, CFS exposure led to an *increase* in dltC expression compared to control, while LA treatment left it largely unchanged. This means that under CFS, *S. aureus* actively reinforces its CAMPs resistance machinery, the very pathway that is compromised when *dlt* genes are knocked out in experimental infections.

Interestingly, the induction of *dltC* under CFS could reflect bacterial sensing of antimicrobial peptides or related stress signals present in the supernatant. The *dlt* operon is known to be under the control of the GraRS two-component system, which responds to cationic antimicrobial peptides and activates protective modifications of the cell envelope [108,109]. This suggests that components of the CFS may mimic or contain CAMPs-like activity, thereby eliciting a stronger defensive response than LA.

- **Chaperonins and Protein Folding Stress**

Proteins involved in protein folding and stress mitigation, including GroES (+1.29) and GrpE (+1.08) (Supplementary Figure S2), were more abundant in the CFS condition compared to LA. These proteins form essential components of the GroEL/GroES and DnaK/GrpE chaperone systems, also known as heat shock protein (Hsp) 70, which assist in the folding and refolding of proteins under cellular stress conditions such as oxidative imbalance, temperature shifts, or protein misfolding overload [110].

Although GroEL and DnaK themselves were not detected in our dataset, the increased expression of their known co-chaperones supports the activation of proteostasis mechanisms in response to CFS. This, therefore, suggests that CFS may trigger a more robust cytoplasmic stress response than LA alone, potentially due to the presence of metabolites or signalling molecules capable of inducing oxidative or metabolic stress defences.

- **Oxidative Stress and Translation**

In the comparison between CFS and LA, an important distinction is the increased abundance of the superoxide dismutases SodA and SodM (1.34 and 1.39, respectively) (Supplementary Figure S2) specifically under CFS treatment. As previously discussed, these enzymes are central to the oxidative stress defence of *S. aureus*, with SodA providing Mn-dependent detoxification of ROS during acid stress and SodM offering additional flexibility by functioning with either manganese or iron, particularly under manganese limitation imposed by host nutritional immunity [77,78]. The fact that CFS, but not LA, induces both SodA and SodM suggests that CFS provokes a broader and more integrated stress response. Thus, the induction of SodA and SodM under CFS highlights its ability to engage more physiologically relevant stress responses than LA alone, which appears insufficient to trigger this full defence network, reinforcing the broader impact of CFS on *S. aureus* physiology.

In addition to oxidative stress defences, several proteins involved in translation and transcription, specifically RpmC (50S ribosomal protein L29), RplX (50S L24), RpsP (30S S16), and the RNA polymerase delta subunit RpoE (Supplementary Figure S2), were detected under CFS treatment but not under LA. These differences, though subtle in fold change, point to a broader disturbance in protein synthesis machinery under CFS exposure. Many other proteins in this functional category showed a similar expression trend. This supports the idea that CFS induces a more extensive stress response, likely reflecting a stronger disruption of cellular homeostasis.

- **Cell Wall Remodelling and Virulence Factors**

The differential expression of Spa (+1.11) and Sbi (−1.28) in CFS versus LA highlights how *S. aureus* adjusts its immune evasion strategy in response to different stress profiles. Spa (Protein A) (Supplementary Figure S2), which binds host immunoglobulins, was more highly expressed in cells exposed to CFS. Since Spa does not require WTAs for cell wall anchoring [87], it may serve as an alternative surface virulence factor when WTA biosynthesis is impaired. While both LA and CFS suppress WTA-related proteins, the stronger upregulation of Spa in CFS-treated cells might reflect a greater degree of envelope stress, triggering *S. aureus* to compensate by increasing expression of WTA-independent proteins. In contrast, Sbi, a SaeRS-regulated immune evasion protein, was more strongly decreased under CFS than LA. Since acid stress and other environmental pressures repress the SaeRS system, this suggests that CFS exerts a more potent or diverse inhibitory effect on this regulatory pathway. Together, these findings support the idea that *S. aureus* adapts its virulence expression in a stress-specific manner, with CFS triggering a distinct response.

### 3.3. Contextualisation, Limitations, and Clinical Implications

To the best of our knowledge, this is the first study employing global proteomic analysis to investigate the changes in the bacterial proteome response of pathogenic bacteria, specifically *P. aeruginosa* and *S. aureus*, to probiotic-derived metabolites, including LA and CFS. Previous studies have primarily applied different approaches, such as transcriptomics [111], targeted metabolomics [112], or secretome proteomics, which focus on the bioactive molecules secreted by probiotic strains [113].

Mehboudi and colleagues demonstrated through gene expression analysis that probiotic cell-free metabolites downregulate antibiotic resistance-associated genes, particularly efflux pumps, in *P. aeruginosa* [111]. This finding is consistent with our previous observations, where exposure to CFS did not promote resistance development, in contrast to traditional antibiotics [33]. Nevertheless, further studies integrating these complementary methods could be useful to comprehensively assess how probiotic-derived treatments influence both resistance and adaptive responses.

Similarly, Myo and colleagues used metabolomics to identify organic acids, particularly LA, as key antimicrobial factors against *S. aureus* [112]. In line with these findings, our data show that LA causes interesting proteomic changes that could affect pathogen physiology, while also leaving open the possibility that other bioactive compounds present in CFS contribute to the overall antimicrobial effect.

Díaz and colleagues showed that lipophilic fractions from probiotic supernatants can inhibit QS and biofilm formation in *P. aeruginosa*, suggesting that non-lactic acid bioactive compounds may also contribute to the effects of CFS [113]. In our data, however, the strong metabolic influence of LA may overshadow these specific anti-virulence effects, highlighting the value of complementary analyses such as lipidomics to uncover such components.

Unlike these previous studies, which primarily characterise probiotic outputs, our work directly investigates the proteomic changes within the pathogens themselves. While LA appears to drive much of the observed response, other metabolites may contribute to the antimicrobial effects. Future studies integrating lipidomics, metabolomics, and functional assays may capture complementary mechanisms, especially those involving membrane interactions or non-protein-mediated pathways. Such multi-omics approaches will be essential to fully understand the complexity of probiotic-pathogen interactions.

This study presents some limitations that should be taken into account when interpreting the results. All experiments were conducted under controlled in vitro conditions, which inevitably simplify the complexity of host-associated environments. The biological activity of probiotic-derived metabolites such as CFS is known to vary substantially depending on the surrounding matrix, the composition of the resident microbiota, and the physiological state of the host. Some human studies have shown that responses to probiotic-derived interventions differ widely across individuals, reflecting variations in strain specificity, formulation, baseline microbiome composition, and immune status [114]. Moreover, LFQ proteomics measures relative changes in protein abundance and does not directly establish functional causality. The proteomic patterns described here therefore represent testable hypotheses that will require validation through targeted functional assays to determine their mechanistic and physiological relevance.

Despite these constraints, the findings obtained provide novel insights with direct translational relevance. The modulation of pathways involved in membrane homeostasis, energy metabolism, oxidative-stress responses, and virulence highlights processes essential for bacterial survival during infection, and that can be weakened by LA and CFS. These observations support the potential development of postbiotic preparations as topical applications. A growing body of literature has already explored the use of postbiotics or probiotic-derived metabolites in clinically relevant contexts, ranging from wound management [17,33] to respiratory conditions such as cystic fibrosis [34,115]. Such settings are particularly suited for local delivery, where the application of metabolite-based interventions may enhance efficacy while limiting systemic exposure.

Looking ahead, further work is required before these postbiotic strategies can progress toward therapeutic translation. Although the pathogenic responses identified here are encouraging, additional studies in suitable in vivo models are essential to assess their impact on the local microbiota, evaluate safety in physiologically relevant environments, and confirm whether the proteomic responses observed in vitro are maintained under host-associated conditions. Addressing these aspects will be crucial to defining the therapeutic potential, feasibility, and safety profile of postbiotic-based interventions.

## 4. Materials and Methods

### 4.1. Bacterial Strain and Culture Conditions

*P. aeruginosa* used throughout this study (PaCF1), is a non-mucoid clinical isolate, part of a collection of strains from the Microbiology laboratory of the University of Pisa. It was originally isolated from the sputum of a chronically infected cystic fibrosis patient [34]. *L. rhamnosus* was isolated from a commercially available Italian product as previously described [34]. Species identification for PaCF1 and *L. rhamnosus* was carried out using MALDI-TOF mass spectrometry (Bruker Daltonics, Bremen, Germany). The *S. aureus* strain used in this work is the reference strain ATCC 33591.

Cultures of *P. aeruginosa* and *S. aureus* were maintained on Tryptone Soy Agar (Oxoid, Basingstoke, Hampshire, UK), while *L. rhamnosus* was maintained on De Man–Rogosa–Sharpe agar (Oxoid, Basingstoke, Hampshire, UK). For stock culture preparation, bacterial strains were grown in Tryptic Soy Broth (Oxoid, Basingstoke, Hampshire, UK) for *P. aeruginosa* and *S. aureus*, or in De Man–Rogosa–Sharpe Broth (Oxoid, Basingstoke, Hampshire, UK) for *L. rhamnosus*, until the late logarithmic phase. Bacteria were pelleted, supernatants were removed, and the cells were resuspended in fresh medium supplemented with 30% glycerol (Sigma-Aldrich, Darmstadt, Germany). The suspensions were divided into aliquots and stored at −80 °C until use.

### 4.2. Preparation of CFS and LA Solution

*L. rhamnosus* was grown in De Man–Rogosa–Sharpe broth under shaking conditions for 48 h. Afterwards, the cultures were centrifuged at 4000× *g* for 10 min, and the supernatants were filtered to sterilisation using 0.22 μm filters (Millipore, Billerica, MA, USA). The resulting CFS were aliquoted and stored at −20 °C until needed.

A LA solution was prepared by adding LA sodium salt (Sigma-Aldrich, St. Louis, MO, USA) to MRSB medium to reach a final concentration of 125 mM, corresponding to the concentration previously detected in the CFS, as previously described [18].

### 4.3. Extraction and Purification of Protein from P. aeruginosa or S. aureus Treated with CFS or LA

An overnight culture of *P. aeruginosa* or *S. aureus* was grown in TSB and then diluted 1:200 into 10 mL of fresh TSB, reaching an estimated starting inoculum of ~10^7^ CFU/mL. These cultures contained either freshly prepared CFS, LA or MRSB at the concentrations described as follows. Preliminary growth assessments were performed by monitoring the optical density at 600 nm (OD_600_) over a 6 h incubation period under the selected culture conditions, which allowed us to identify the sub-inhibitory concentrations used for the proteomic analysis. Both the CFS and LA treatments were added at a sub-MIC dilution of 1:10 for *P. aeruginosa* and of 1:8 for *S. aureus*. LA was added at the same concentration present in the CFS diluted 1:10 and 1:8, corresponding to 12.5 mM for *P. aeruginosa* and 15.6 mM for *S. aureus*, respectively. The control condition was added with the same amount of MRSB. The analysis was carried out in triplicate for both *P. aeruginosa* and *S. aureus* on the three different sample groups (CTRL, LA, and CFS) for a total of 9 samples for each bacterial species. Cultures were incubated at 37 °C on an orbital shaker (Innova 4000, New Brunswick Scientific, Edison, NJ, USA) at 150 rpm for 6 h, allowing the bacteria to reach the late exponential phase. Following incubation, proteins from the bacterial cell lysates were processed for LC–MS/MS analysis following the workflow described by Margalit and colleagues [116], with minor adaptations as detailed below. The bacterial cells were collected by centrifugation and washed twice with PBS to remove extracellular proteins and any residual proteins originating from the CFS added at the beginning of the experiment. The washed cells were then resuspended in 1 mL of Lysis Buffer (8 M urea, 2 M thiourea, 0.1 M Tris-HCl, pH 8.0) (Sigma-Aldrich, Darmstadt, Germany) containing protease inhibitors (aprotinin, leupeptin, PMSF, and pepstatin A) and lysed with an ultrasonic homogeniser (Bendelin Senopuls, Berlin, Germany). Protein concentration in the cell lysates was determined using the Bradford assay (Bio-Rad, Hercules, CA, USA), in order to take an appropriate volume of each lysate to obtain 100 µg of total protein, which was precipitated overnight with cold acetone (1:6 ratio). The resulting pellets were resuspended in denaturing buffer (8 M urea, 2 M thiourea, 0.1 M Tris-HCl, pH 8.0) prepared with Milli-Q water (Merck Millipore, Darmstadt, Germany). Post-precipitation protein quantification was conducted using the Qubit system (Invitrogen, Carlsbad, CA, USA) in accordance with the manufacturer’s instructions. Proteins were reduced with 0.5 M DTT (Sigma-Aldrich, Darmstadt, Germany) at 56 °C for 20 min, followed by alkylation with 0.55 M iodoacetamide (Sigma-Aldrich, Darmstadt, Germany) in the dark at room temperature. Digestion was performed with Protease Max Surfactant Trypsin Enhancer (1% *w*/*v*, Promega, Madison, WI, USA) and Sequence Grade Trypsin (Life Technologies, Waltham, MA, USA) at 37 °C for 18 h. Digestion was terminated with 100% Trifluoroacetic acid (TFA) (Thermo Fisher Scientific, Waltham, MA, USA), and peptides were purified using C18 Spin Columns (Pierce, Life Technologies, Waltham, MA, USA) following the manufacturer’s protocol. The purified peptides were dried in a SpeedVac concentrator (Savant DNA130, Thermo Fisher Scientific, Waltham, MA, USA) until mass spectrometry analysis.

### 4.4. LFQ Analysis of P. aeruginosa and S. aureus Cell Lysates Treated with CFS: Preparation and Data Analysis

Lyophilised proteins from the cell lysates of *P. aeruginosa* and *S. aureus* were resuspended to a final concentration of 300 ng/μL, of which 2 μL was loaded onto the Q-extractive mass spectrometer (HPLC-MS/MS) (Thermo Fisher Scientific, Waltham, MA, USA) using a 133 min reverse-phase gradient. Quantitative proteomic analysis of the *P. aeruginosa* and *S. aureus* cell lysates treated with CFS was performed using the software MaxQuant v. 2.6.4.0. *P. aeruginosa* and *S. aureus* reference proteome obtained from a UniProtKB (identifier 47715, protein count 2732, https://www.uniprot.org/taxonomy/47715, accessed on 5 November 2024) obtained from UniProt [117]). UniProt-SWISS-PROT database to identify proteins (9647 entries, downloaded July 2022). Trypsin/P was specified as the proteolytic enzyme, allowing up to two missed cleavages. The minimum peptide length was set to seven amino acids, and the maximum peptide mass was limited to 4600 Da. The false discovery rate (FDR) was controlled at 1% at both the peptide and protein levels. The “*match between runs*” feature was not applied; peptide identifications were therefore based exclusively on MS/MS evidence within each individual LC–MS/MS run.

Total LFQ intensities obtained from the MaxQuant analysis were imported into Perseus 2.1.2.0 [118]. The data were filtered to remove potential contaminants, reverse hits, and proteins identified only by site. Subsequently, the data were Log_2_-transformed, and the distribution normality was assessed (Supplementary Figure S1). Samples were then filtered based on the “Valid Values 75%” criterion (“In at least one group”), and missing values were imputed. PCA was performed, and the number of significant principal components was determined using a scree plot (Supplementary Figure S2). Matrices were then exported to produce volcano plots on GraphPad 9.0. Heatmap analysis was performed in Perseus following an ANOVA-based multiple sample test. Significant features were filtered based on ANOVA significance values, and Z-score normalisation was applied prior to heatmap generation. Protein–protein interaction networks were constructed using STRING version 12.0 (https://string-db.org/, accessed on 16 January 2025). Statistically significant and differentially abundant (SSDA) proteins were input based on their gene identifiers obtained from UniProt for *P. aeruginosa* PAO1 and *S. aureus* NCTC 8325. A stringent interaction confidence threshold of 0.7 was applied, and any unconnected nodes were excluded from the final network visualisations comparing treatment and control conditions. Node colouring was based on clusters identified using the Markov Cluster Algorithm (MCL) implemented within the STRING platform.

### 4.5. Data Availability

The proteomics raw data and MaxQuant search output files have been deposited to the ProteomeXchange Consortium [119] via the PRIDE partner repository with the dataset identifier PXD069979.

## 5. Conclusions

This study provides the first global proteomic overview of how probiotic-derived metabolites, including LA and CFS, influence the intracellular physiology of *Pseudomonas aeruginosa* and *Staphylococcus aureus*.

In *P. aeruginosa*, LA emerged as the main driver of proteomic alterations, promoting metabolic adjustments and repression of systems related to adhesion and secretion, consistent with reduced virulence potential. The proteomic profiles of cells exposed to CFS and LA were largely similar, suggesting that acidification represents the dominant stress in this species.

In contrast, *S. aureus* displayed a broader and more distinct response between the two treatments, including extensive cell wall remodelling associated with WTA suppression and altered abundance of autolysin-related proteins, together with the reduction in SaeRS-regulated virulence factors. These findings indicate that, in *S. aureus*, additional bioactive components present in the CFS may contribute to the observed effects beyond those caused by LA alone.

In summary, LA defines the core adaptive response to probiotic stress, while additional components in the CFS appear to extend this response toward cell envelope maintenance and virulence modulation. This layered adaptation reflects how pathogens dynamically adjust to complex probiotic environments.

## Figures and Tables

**Table 1:** Top 10 most and least abundant significant proteins in *P. aeruginosa* in the comparison between *L. rhamnosus* cell-free supernatant (CFS) and control.

Gene Names	Protein IDs	Protein Names	−Log_10_ (*p*-Value)	Log_2_ (Fold Change)
nirM	P00099	Cytochrome c-551 (Cytochrome C8)	4.05	5.38
ureG	Q9HUS0	Urease accessory protein UreG	4.05	4.10
cyoA	Q9I427	Cytochrome bo(3) ubiquinol oxidase subunit 2	3.48	4.09
speE2	Q9HV34	Polyamine aminopropyltransferase 2/Spermidine synthase 2 (PAPT 2/SPDS 2)	4.55	3.81
PA0805	Q9I5D3	DUF3509 domain-containing protein	3.85	3.56
speH	Q9HV35	S-adenosylmethionine decarboxylase proenzyme (AdoMetDC) (SAMDC)	3.44	3.55
PA0529	Q9I607	MOSC domain-containing protein	3.64	2.90
PA0531	Q9I605	Probable glutamine amidotransferase	3.49	2.70
gltB	Q9HUD5	Glutamate synthase large chain	1.91	2.64
cyoB	Q9I426	Cytochrome bo(3) ubiquinol oxidase subunit 1	1.32	2.40
PA3688	Q9HXV2	Pilin assembly protein	2.42	−3.05
phzD2 phzD	P0DPC1	Phenazine biosynthesis protein PhzD2 (Isochorismatase)	2.17	−3.06
fabH	Q9HYR2	Beta-ketoacyl-[acyl-carrier-protein] synthase III	2.96	−3.16
phzG1	G3XCV4	Probable pyridoxamine 5′-phosphate oxidase	2.54	−3.31
gbuA	Q9I3S3	Guanidinobutyrase	2.10	−3.46
pcrV P	G3XD49	Type III secretion protein PcrV	4.14	−3.52
phzB1	O69753	Phenazine biosynthesis protein PhzB1	2.50	−3.61
PA5217	Q9HTX3	Probable binding protein component of ABC iron transporter PA5217	2.27	−3.86
phzF1/phzF2	O69754	Phenazine/pyocyanine biosynthesis protein PhzF	3.16	−5.17

**Table 2:** Top 10 most and least abundant significant proteins in in *S. aureus* in the comparison between *L. rhamnosus* cell-free supernatant (CFS) and control.

Gene Names	Protein IDs	Protein Names	−Log_10_ (*p*-Value)	Log_2_ (Fold Change)
sceD	Q2FWF8	Probable transglycosylase SceD	2.68	5.39
SAOUHSC_02576	Q2G1W1	-	2.99	3.80
SAOUHSC_02855	Q2FV81	-	3.87	3.58
sspP	Q2G2R8	Staphopain A	3.96	3.46
lytM	O33599	Glycyl-glycine endopeptidase LytM	3.47	3.41
ssaA	Q2FV55	Staphylococcal secretory antigen SsaA	3.90	3.19
rpmJ	Q2FW29	50S ribosomal protein L36	3.70	3.00
SAOUHSC_03045	Q2FUQ9	-	3.04	2.96
rpmD	P0A0G2	50S ribosomal protein L30	4.76	2.81
SAOUHSC_00819	Q2G009	-	3.27	2.74
adh	Q2G0G1	Alcohol dehydrogenase	4.42	−2.18
saeR	Q2G2G2	Response regulator SaeR	3.00	−2.21
SAOUHSC_01114	Q2FZB8	-	5.18	−2.42
coaW	Q2FWC7	Type II pantothenate kinase	4.21	−2.44
SAOUHSC_02820	Q2FVB4	-	3.99	−2.49
mfd	Q2G0R8	Transcription-repair-coupling factor	3.01	−2.62
SAOUHSC_00717	Q2G2G0	-	4.78	−3.23
ald1	Q2FYJ2	Alanine dehydrogenase 1	3.21	−3.30
tdcB	Q2FYJ3	L-threonine dehydratase catabolic TdcB	4.30	−3.36
sbi	Q2FVK5	Immunoglobulin-binding protein Sbi	3.44	−3.92

## Data Availability

The mass spectrometry proteomics data have been deposited to the ProteomeXchange Consortium via the PRIDE [119] partner repository with the dataset identifier PXD069979.

## Supplementary Materials

The following supporting information can be downloaded at: https://www.mdpi.com/article/10.3390/antibiotics14121271/s1, Figure S1: Growth assessment of *P. aeruginosa* and *S. aureus* exposed to the selected inhibitory and sub-inhibitory dilutions of CFS; Figure S2: Protein Interaction Network analysis of proteins increased or decreased in abundance in *P. aeruginosa* and *S. aureus* in the comparison between CFS- and LA-treated conditions; Table S1: Statistically significant and differentially abundant proteins list of *P. aeruginosa* in the comparisons between CFS, LA and control; Table S2: Statistically significant and differentially abundant proteins list of *S. aureus* in the comparisons between CFS, LA and control.

## Abbreviations

ADI	Arginine deiminase pathway
ATCC	American Type Culture Collection
CAMPs	Cationic antimicrobial peptides
CCR	Carbon catabolite repression
CF	Cystic fibrosis
CFS	Cell-free supernatant
COX-2	Cyclooxygenase-2
CTRL	Control condition
DTT	Dithiothreitol
FASII	Fatty acid synthesis type II pathway
IL-6	Interleukin-6
iNOS	Inducible nitric oxide synthase
LA	Lactic acid
LFQ	Label-free quantitative
LPXTG	Leucine–proline–X–threonine–glycine motif
LPS	Lipopolysaccharide
MALDI-TOF	Matrix-Assisted Laser Desorption/Ionisation Time-of-Flight
MCL	Markov Cluster Algorithm
MIC	Minimum inhibitory concentration
mRNA	Messenger RNA
MRSA	Methicillin-resistant *Staphylococcus aureus*
MRSB	Man, Rogosa and Sharpe Broth
PBS	Phosphate-buffered saline
PCA	Principal component analysis
PMSF	Phenylmethylsulfonyl fluoride
QS	Quorum sensing
ROS	Reactive oxygen species
RPM	Revolutions per minute
SSDA	Statistically significant differentially abundant (proteins)
STRING	Search Tool for the Retrieval of Interacting Genes/Proteins
T3SS	Type III secretion system
TFA	Trifluoroacetic acid
TNF-α	tumour necrosis factor alpha
TSB	Tryptic Soy Broth
WHO	World Health Organisation
WTA	Wall teichoic acid
