# Supplementary figures and images for: Proteomic Analysis of the Differential Response of Pseudomonas aeruginosa and Staphylococcus aureus to Lacticaseibacillus rhamnosus Cell-Free Supernatant and Lactic Acid
Source: Antibiotics (Basel). 2025 Dec 15;14(12):1271. doi: 10.3390/antibiotics14121271 (PMC12730115; doi:10.3390/antibiotics14121271)

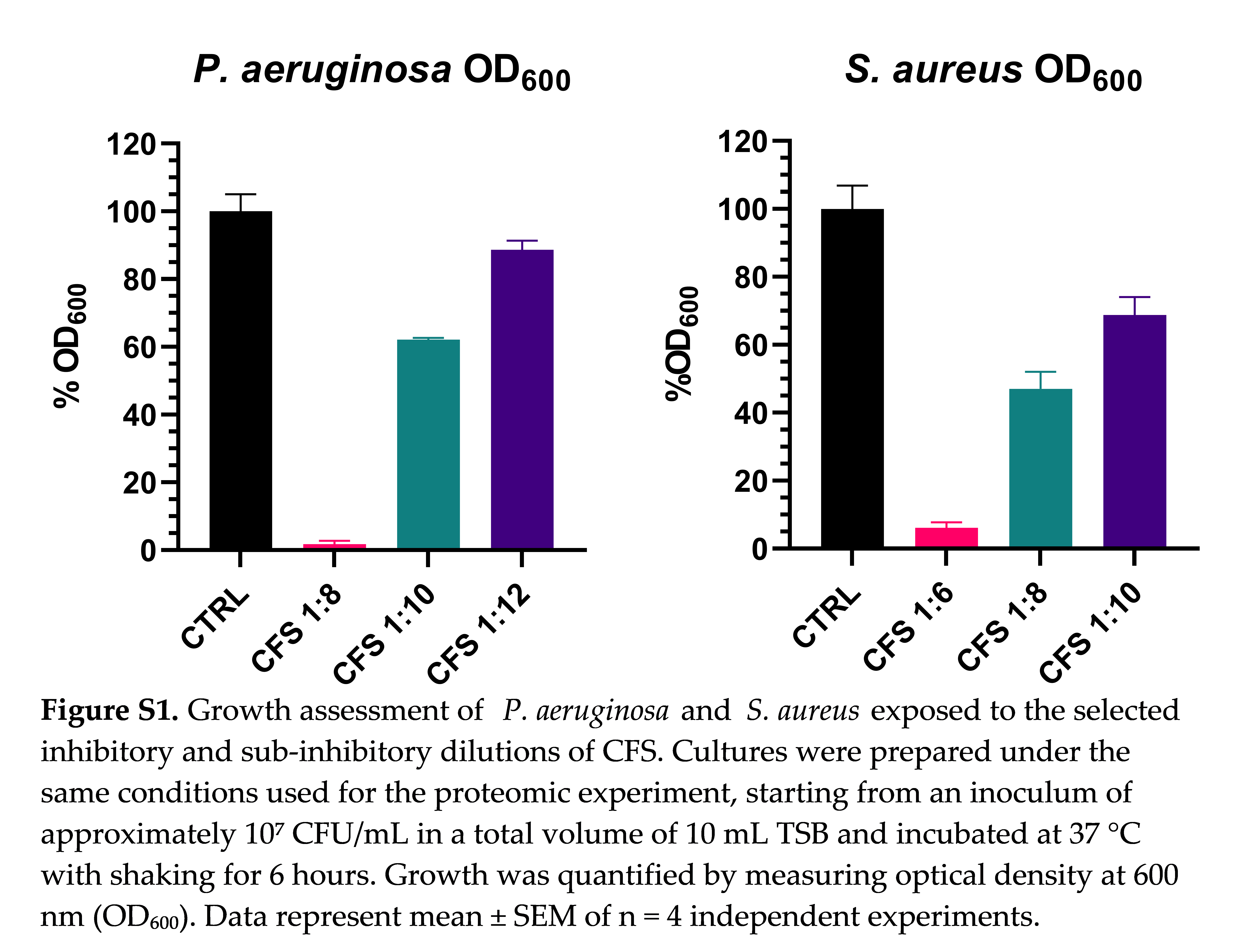

Supplement: Supplementary file 1 [file antibiotics-14-01271-s001.zip › Figure S1.png]

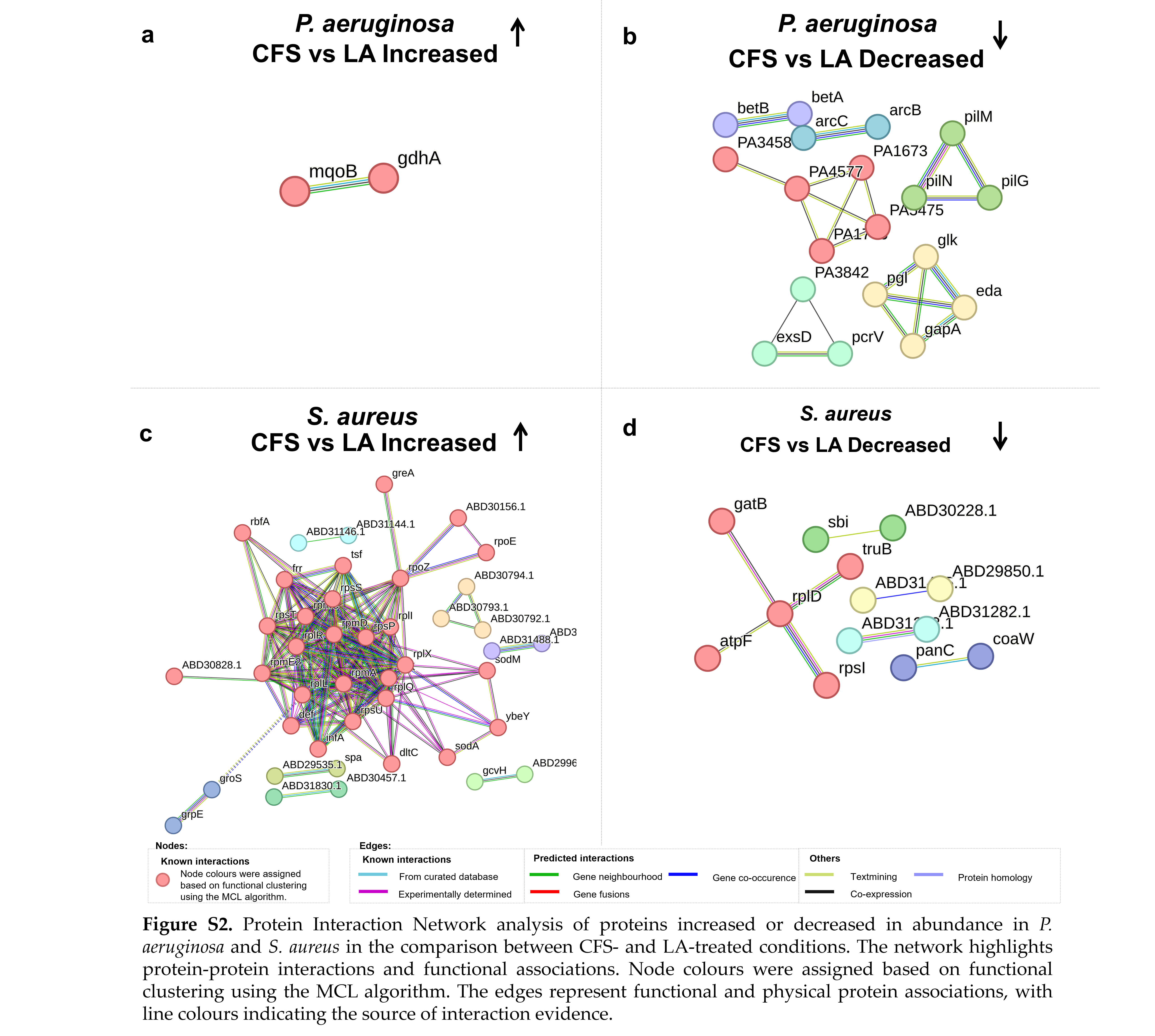

Supplement: Supplementary file 1 [file antibiotics-14-01271-s001.zip › Figure S2.PNG]
